# Characterization and treatment of ischemic stroke in patients with infective endocarditis: a meta‐analysis

**DOI:** 10.1186/s42466-026-00542-z

**Published:** 2026-10-26

**Authors:** Nils Werring, Nadine Bonberg, Andreas Heidenreich, Carolin Beuker, Greta Noblejas-Sanchez, Michelle Oswald, Georg Royl, Tobias A. Wagner-Altendorf, Tanja Zeller, Thomas Stiermaier, Ingo Eitel, Alexander Katalinic, Heike Minnerup, Jens Minnerup

**Affiliations:** 1 University of Luebeck, Department of Neurology, Luebeck, Germany; 2 Institute of Epidemiology and Social Medicine, University of Muenster, Muenster, Germany; 3 University of Luebeck, Institute of Social Medicine and Epidemiology, Luebeck, Germany; 4 Department of Neurology with Institute of Translational Neurology, University of Muenster, Muenster, Germany; 5 University of Luebeck, Institute of Cardiogenetics, Luebeck, Germany; 6 University of Luebeck, Medical Clinic II, University Heart Center Luebeck, Luebeck, Germany; 7 partner site North, DZHK (German Center for Cardiovascular Research), Lübeck, Germany

**Keywords:** Endocarditis, Bacterial, Stroke, Thrombectomy, Embolism, Meta-analysis

## Abstract

**Background:**

Ischemic stroke is a common and serious complication of infective bacterial endocarditis. This study aimed to characterize the frequency, clinical and pathological features, treatment strategies, and outcomes of patients with stroke due to infective endocarditis.

**Methods:**

We systematically searched PubMed from database inception to October 2022 for studies reporting on stroke in patients with infective endocarditis and performed a meta-analysis.

**Results:**

A total of 122 studies comprising 270,211 patients with infective endocarditis were included. Ischemic stroke occurred in 21% and hemorrhagic stroke in 5% of patients. Stroke was observed in 32% of patients with mitral valve endocarditis, 25% with aortic valve, and 11% with tricuspid valve endocarditis. Stroke was associated with significantly higher mortality (OR 2.35; 95%CI 1.74–3.16). Staphylococcus and Streptococcus species were most frequently associated with stroke (33% and 25%, respectively). The risk of stroke did not differ significantly between patients with and without pre-existing antithrombotic therapy (37% vs. 24%; OR 1.24, 95%CI 0.75–2.06). Successful reperfusion was achieved in 80% of patients undergoing mechanical thrombectomy, although mortality remained high at 49%.

**Conclusions:**

Stroke is a common complication of infective bacterial endocarditis and has a substantial impact on patient outcomes. It occurs most frequently in patients with mitral valve endocarditis and in those with infections caused by staphylococcal pathogens. Stroke risk was not reduced in patients with pre-existing antithrombotic therapy, supporting discontinuation where no other indication exists. Mechanical thrombectomy in patients with endocarditis-related large vessel occlusion achieves high recanalization rates; however, overall clinical outcomes remain poor in this population.

**Supplementary Information:**

The online version contains supplementary material available at https://doi.org/10.1186/s42466-026-00542-z.

## Introduction

Bacterial infective endocarditis (IE) is a life-threatening disease with an estimated incidence of 13.8 per 100,000 individuals, which has steadily increased over the past three decades [1]. In parallel, IE related mortality has increased despite substantial advances in antibiotic therapy [1]. Ischemic and hemorrhagic stroke are among the most common and severe complications of IE, occurring in 15–40% of affected patients [2–4].

However, despite the high clinical relevance of stroke in IE, important questions regarding its characteristics and optimal management remain unresolved. Mechanical thrombectomy is increasingly performed in patients with IE-related large vessel occlusion; however, its safety, feasibility and clinical benefit have not yet been clearly established in this population [5]. In addition, the use of antithrombotic therapy in patients with IE remains controversial, as the potential benefits in preventing ischemic events must be weighed against an increased risk of bleeding complications [6, 7].

The aim of the present study was to characterize the clinical and laboratory features, as well as feasibility and outcomes of acute treatments of patients with IE complicated by ischemic and hemorrhagic stroke. To this end, we conducted a meta-analysis of published studies reporting cohorts and case series of patients with IE and stroke, including data from our own cohort.

## Methods

### Standard protocol approvals, registrations, and patient consents

The study was approved by the local ethics committee (AZ 2025-574-f-S) and conducted in accordance with the Declaration of Helsinki. Written informed consent was waived owing to the retrospective study design based on the review of patient medical records.

### Study oversight

We conducted a systematic literature search and meta-analysis of observational studies reporting ischemic and hemorrhagic stroke in patients with IE. The meta-analysis assessed the following patient characteristics and outcomes: overall frequency of stroke, mortality, stroke frequency by pathogen and pathogen distribution among stroke cases, stroke frequency by affected cardiac valve and distribution of affected valves among stroke cases, and prevalence and outcome of patients with previous antiplatelet or anticoagulant use. For studies reporting mechanical thrombectomy in patients with IE and ischemic stroke, additional characteristics were analyzed in combination with data from our own cohort including the proportion of successful reperfusion, frequency of intracranial hemorrhages, and in-hospital mortality.

### Literature search and study selection

We performed a systematic literature search and meta-analysis according to the Preferred Reporting Items for Systematic Reviews and Meta-Analyses (PRISMA) and Meta-analysis of Observational Studies in Epidemiology (MOOSE) guidelines. We searched PubMed using the terms “*endocarditis*” AND (“*stroke*” OR “*ischemia*” OR “*infarction*”) for articles published up to October 1, 2022. Eligible studies were peer-reviewed clinical studies published in English that included adult patients (≥ 18 years) diagnosed with infective endocarditis and reported on early stroke outcomes. Exclusion criteria were: (1) infective endocarditis associated with immunocompromised conditions such as HIV infection or solid organ or hematopoietic stem cell transplantation; (2) stroke occurring as complication of cardiac surgery; and (3) autopsy studies. To reduce systematic bias stroke studies reporting fewer than three patients with stroke were excluded as were studies reporting only on congenital heart disease or intravenous drug use. Two investigators (NW and CB) independently performed the literature search, and each screened studies for eligibility. Disagreements were resolved by consensus after joint review of the relevant publications (NW and CB) or, if necessary, by consultation of a third reviewer (JM). For studies or data published more than once (i.e., duplicates), only the report providing the most comprehensive and informative data was included. A formal risk of bias assessment was not performed.

### Data extraction

We extracted data on the following variables: year of publication, sample size, study period, study location, study design, initial clinical presentation, onset and type (ischemic or hemorrhagic) of stroke, causative pathogen, affected cardiac valves, prior use of anticoagulant or antiplatelet therapy, and clinical outcomes.

### Participants of the single-center cohort study

In addition to the meta-analysis, we included 18 adult patients diagnosed with stroke due to IE admitted to the Neurological Department, University Hospital of Münster, Münster, Germany between 2010 and 2022. All patients met the following diagnostic criteria: (1) definite infective endocarditis according to the modified Duke Criteria, (2) ischemic or hemorrhagic stroke attributable to IE, and (3) attempted mechanical thrombectomy.

All patients were followed until death or hospital discharge, whichever occurred first. Functional outcome was assessed using the modified Rankin scale (mRS) and categorized as follows: no symptoms (0), no significant disability (1), slight disability (2), moderate disability (3), moderately severe disability (4), severe disability (5), and death (6). Successful reperfusion was defined as mTICI (Thrombolysis in cerebral infarction) scores 2b or 3.

### Statistical analysis

Meta-analyses of proportions were conducted using the *metaprop* function from the *meta* package [8] in R, applying a generalized linear mixed model (GLMM). All analyses were performed with a random-effects model, which was considered the most appropriate and conservative approach given the expected clinical and methodological heterogeneity between studies. This approach accounts for between-study variability by allowing true effect sizes to vary across studies and results in wider confidence intervals that better reflect real-world uncertainty and reduce overconfidence in estimates [9]. Common effect estimates were additionally calculated as a sensitivity analysis.

Pooled estimates are presented as proportions with 95% confidence intervals. For subgroup analyses, we compared pooled proportions between clinically relevant groups. Differences between subgroup estimates were assessed using a z-test for proportions. Statistical significance was defined as a two-sided p-value < 0.05.

To examine whether stroke incidence differed across valve locations, a mixed-effects meta-regression using the *rma* function from the *metafor* package [10] in R was performed comparing stroke rates in mitral, aortic, and tricuspid valve endocarditis, with the affected valve as a categorical moderator. Pairwise comparisons between affected valves were conducted with Holm correction for multiple testing.

All analyses were performed using R software (version 4.5) [11].

## Results

### Frequency of stroke in patients with infective endocarditis

Of 3,729 records identified through the literature search, 257 studies were assessed for eligibility, of which 122 met the inclusion criteria and were included in the meta-analysis (Suppl. Figure 1). These studies comprised a total of 270,211 unique patients diagnosed with infective endocarditis. All included studies were hospital-based single- and multi-center cohort studies. Supplementary Fig. 2 depicts the geographic distribution of the included studies.

**Fig. 1:**
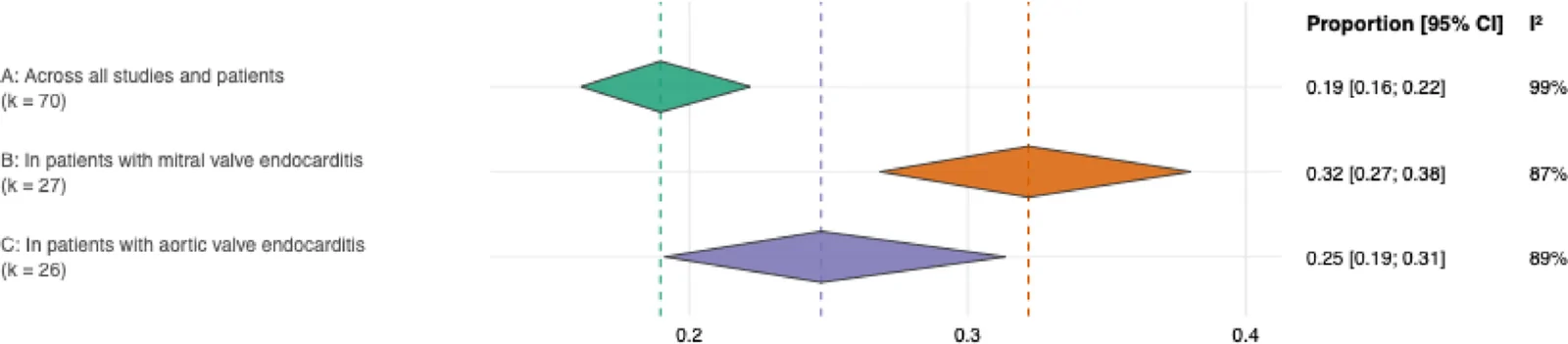
Frequency of stroke in patients with infective endocarditis; A: Across all studies and patients, B: In patients with mitral valve endocarditis, C: In patients with aortic valve endocarditis

Among patients with infective endocarditis, ischemic stroke occurred in 21% (95%CI 17–24) and hemorrhagic stroke in 5% (95%CI 4–7). In studies that did not distinguish between ischemic and hemorrhagic events stroke of unspecified type occurred in 19% (95%CI 16–22) of patients with IE (Fig. 1A, Suppl. Figure 3 A).

### Frequency of affected cardiac valves

Stroke occurred in 17% of patients with native valve endocarditis and 19% of patients with prosthetic valve endocarditis (data not shown). When stratified by valve location, stroke occurred in 32% of patients with mitral valve endocarditis (Fig. 1B, Suppl. Figure 3B), 25% of those with aortic valve endocarditis (Fig. 1C, Suppl. Figure 3 C), 11% of those with tricuspid valve endocarditis (Suppl. Figure 3D), and there were insufficient data on stroke in pulmonary valve endocarditis. In logistic meta-regression, valve location was a significant predictor of stroke incidence (*p* = 0.002). Both mitral valve endocarditis (OR 3.47, 95% CI 1.69–7.13, *p* = 0.002) and aortic valve endocarditis (OR 2.44, 95% CI 1.16–5.00, *p* = 0.03) were associated with higher stroke risk than tricuspid valve endocarditis in pairwise comparisons. The difference between mitral and aortic valve endocarditis was not statistically significant (OR 1.44, 95% CI 0.96–2.15, *p* = 0.08).

### Frequency of pathogens in patients with stroke due to infective endocarditis

**Fig. 2:**
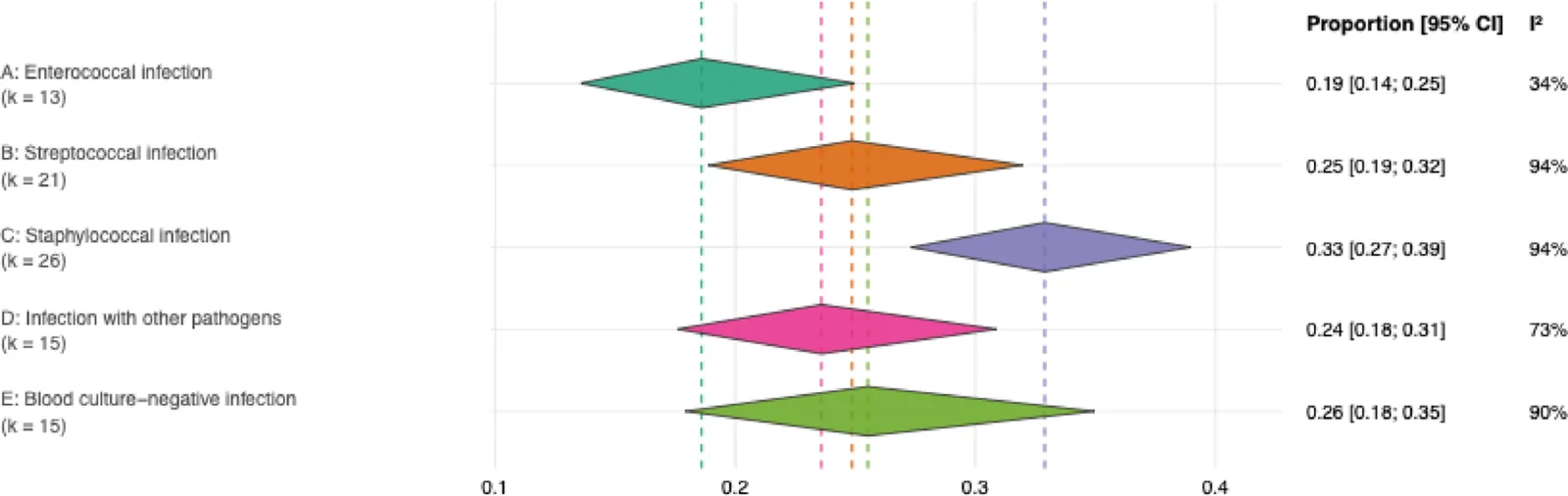
Frequency of stroke in patients with A enterococcal, B streptococcal, C staphylococcal, D others and E blood culture negative infective endocarditis

**Fig. 3:**
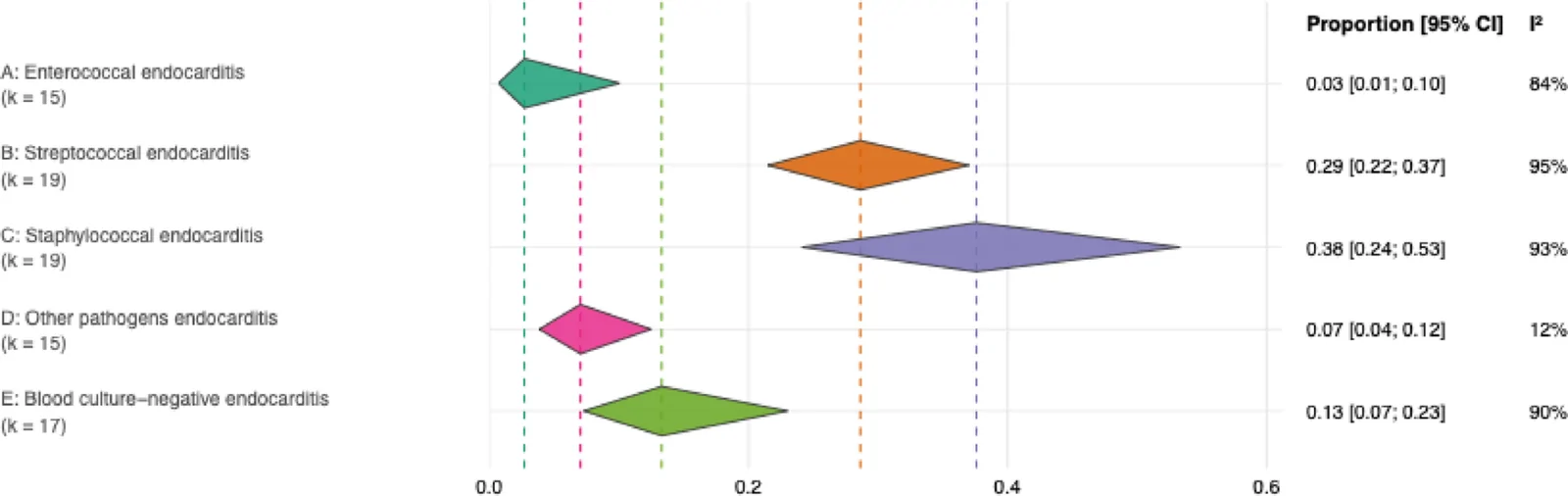
Frequency of A enterococcal, B streptococcal, C staphylococcal, D others and E blood culture negative endocarditis in patients with stroke due to infective endocarditis

Among patients with IE complicated by ischemic or hemorrhagic stroke, stroke frequency stratified by causative pathogen was as follows: *Enterococcus* species, 19% (95%CI 14–25); *Streptococcus* species, 25% (95%CI 19–32); *Staphylococcus* species, 33% (95%CI 27–39); other pathogens, 24% (95%CI 18–31); and blood culture-negative IE, 26% (95%CI 18–35) (Fig. 2, Suppl. Figure 4). The distribution of causative pathogens in patients with stroke due to IE was as follows: *Enterococcus* species 3% (95%CI 1–10), *Streptococcus* species 29% (95%CI 22–37), *Staphylococcus* species 38% (95%CI 24–53), other 7% (95%CI 4–12), and blood culture negative IE, 13% (95%CI 7–23) (Fig. 3, Suppl. Figure 5).

### Frequency of anticoagulation and anti-platelet therapy

**Fig. 4:**
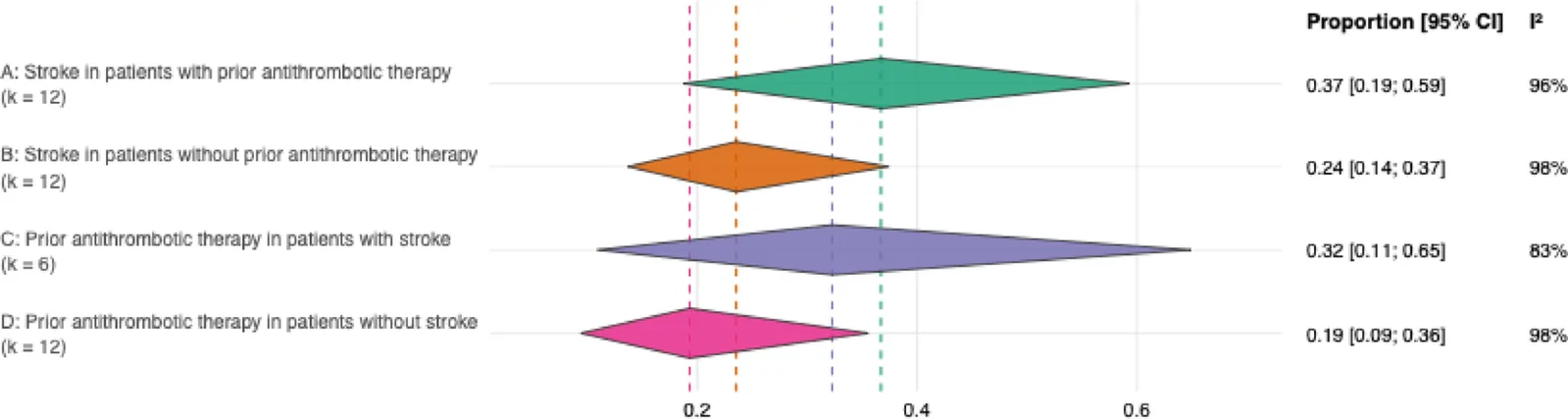
Frequency of stroke in patients with IE A with pre-existing anticoagulation or antiplatelet therapy and B without prior anticoagulation or antiplatelet therapy. Frequency of pre-existing anticoagulation or antiplatelet therapy in patients with IE C with stroke and D without stroke

Stroke occurred in 37% of patients with IE with prior anticoagulant or antiplatelet use and in 24% of those without prior antithrombotic therapy (*p* = 0.27; Fig. 4A and B, Suppl. Figure 6 A and B**)**. Among patients with stroke due to IE, 32% had received prior anticoagulant or antiplatelet therapy, compared with 19% of patients with IE without stroke (*p* = 0.40; Fig. 4C and D, Suppl. Figure 6 C and D).

### Mortality in patients with stroke due to infective endocarditis

**Fig. 5:**
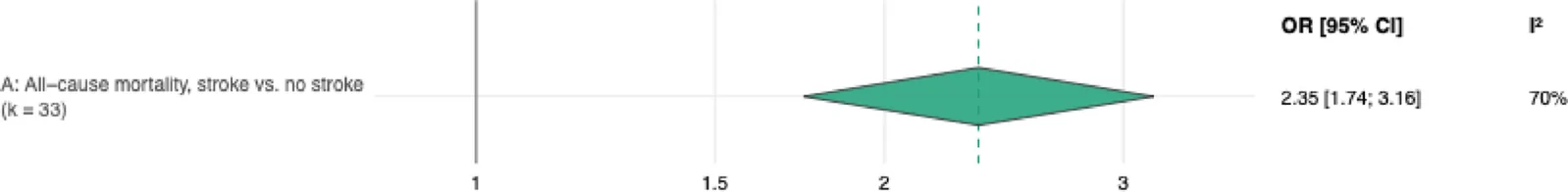
Mortality in patients with stroke due to infective endocarditis

The occurrence of stroke in patients with IE was strongly associated with increased mortality (OR 2.35; 95%CI 1.74–3.16) (Fig. 5, Suppl. Figure 7). Mortality was 22% in patients with IE and stroke compared to 10% in patients with IE without stroke (*p* < 0.001).

### Effects of recanalization in patients with stroke due to endocarditis

**Fig. 6:**
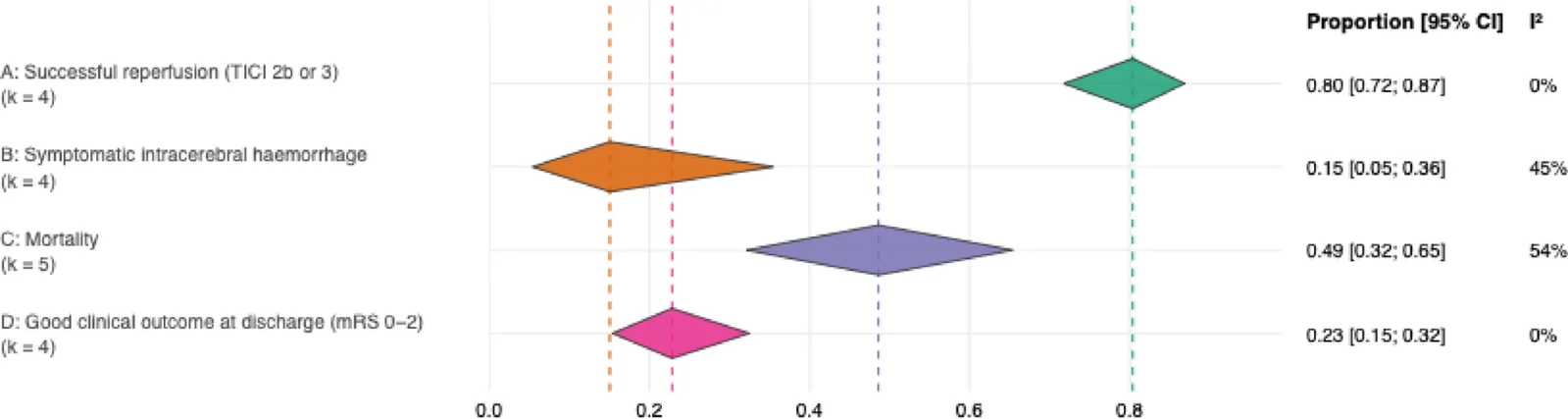
Frequency of A successful reperfusion (TICI 2b or 3), B symptomatic intracerebral hemorrhage, C mortality and D good clinical outcome at discharge (mRS 0–2) in patients with IE and stroke undergoing mechanical thrombectomy

Four studies reporting outcomes on patients with IE and stroke treated with mechanical thrombectomy [12–15] were included and combined with data from our own cohort. In total, 110 patients underwent mechanical thrombectomy. Successful reperfusion was achieved in 80% of patients (95% CI 72–87%, Fig. 6A, Suppl. Figure 8 A). The proportion of symptomatic intracranial hemorrhage was 15% (95% CI 5–36%; Fig. 6B, Suppl. Figure 8B). Overall mortality was 49% (95% CI 32–65; Fig. 6C, Suppl. Figure 8 C), and a good functional outcome (mRS 0–2) was achieved in 23% of patients (95% CI 15–32%; Fig. 6D, Suppl. Figure 8D).

## Discussion

Despite advances in antimicrobial therapy and cardiac surgery, stroke remains one of the most common and prognostically consequential complications of infective endocarditis. This meta-analysis of 122 studies comprising more than 270,000 patients yielded the following main results: First, stroke is common in IE and is a major determinant of outcome. Second, while stroke may occur with any pathogen and any affected cardiac valve, it was most frequently observed in mitral valve endocarditis and in staphylococcal infections. Third, mechanical thrombectomy was associated with high reperfusion rates and a relatively low rate of symptomatic intracranial hemorrhage, but overall outcomes remained poor. Fourth, stroke risk did not differ significantly between patients with and without pre-existing antithrombotic therapy.

Stroke occurred in approximately one in five patients with IE and conferred a substantially worse prognosis, with mortality reaching 22% in patients with stroke versus 10% in those without (OR 2.35, 95% CI 1.74–3.16), confirming the prognostic weight of this complication observed in prior multicenter studies [3, 16]. This twofold increase in mortality likely reflects both the direct consequences of neurological injury and the systemic disease burden of IE itself - patients who sustain embolic stroke tend to have more aggressive infections with larger vegetations, compounding an already high baseline risk independently of the neurological event.

Stroke was more often observed in mitral valve endocarditis and staphylococcal infections, yet occurred across all pathogens and valve locations, including tricuspid valve IE, consistent with prior literature [3, 17]. This broad distribution carries practical diagnostic implications. A positive blood culture in a patient presenting with acute ischemic stroke should prompt consideration of IE regardless of the organism, when the clinical context is compatible, such as prior valve replacement, multi-organ affection, or presence of distinct imaging patterns [3, 18]. This supports maintaining a high index of suspicion rather than restricting diagnostic evaluation to selected “high-risk” microbiological patterns, and looking beyond typical locations such as mitral valve IE, with assessment of possible right-sided IE, where stroke may arise via paradoxical embolism through a patent foramen ovale or other right-to-left shunt [19–21].

With regard to acute ischemic stroke treatment, the harmful effects of intravenous thrombolysis in IE-associated stroke have been well documented and its use is therefore discouraged [22, 23]. In contrast, mechanical thrombectomy is increasingly performed in IE-related large vessel occlusion, and our pooled results indicate that procedural success rates are comparable to those observed in other stroke etiologies [24]. Given that stroke is often presenting symptom of IE and not recognized during the initial diagnostic work-up of acute stroke [25], these findings support considering thrombectomy in eligible patients even when IE is subsequently identified as the underlying cause. The substantially worse overall clinical outcomes after thrombectomy likely reflect the severity of the underlying disease, which is typically required to generate large vegetations capable of causing a large-vessel occlusion amenable to endovascular treatment [26].

The benefit of antithrombotic therapy in IE remains uncertain; both US and European guidance generally discourages initiating antithrombotic therapy in patients without another indication but differ regarding continuation of pre-existing antiplatelet or anticoagulant therapy [6, 7, 27]. Although antithrombotic therapy is biologically plausible for preventing cerebral infarction and has shown in-vitro antithrombotic (and possible antimicrobial) effects, clinical studies have not demonstrated a clear stroke-prevention benefit, particularly when started de novo [28–31]. Furthermore, thrombocytopenia has been associated with increased risk for hemorrhagic complications [32], suggesting that platelet inhibition may compound bleeding risk. In our analysis, stroke occurred more often (though not significantly) among patients receiving pre-existing antithrombotic therapy, which may reflect hemorrhagic complications and confounding by indication due to higher baseline cardiovascular risk in patients prescribed antithrombotics. Taken together, we argue that our data support discontinuation of antithrombotics in patients with IE without any other indication for such medication.

Clinically, the high mortality in IE-associated stroke highlights an urgent need for improved, multidisciplinary strategies that integrate acute stroke pathways with timely IE-directed management.

Our study has several strengths and limitations. Major strengths include the comprehensive and multimodal characterization of stroke in IE and the integration of detailed single-center recanalization data with a large meta-analysis. Key limitations are the predominantly retrospective design of included studies and variability in neurological ascertainment and reporting, including frequent lack of differentiation between ischemic and hemorrhagic strokes, which likely contributed to substantial heterogeneity.

In conclusion, stroke is a common complication of IE, occurs across all causative pathogens and valve locations, and carries a substantially increased risk of death. Mechanical thrombectomy achieves high recanalization rates but does not translate into consistently good clinical outcomes, reflecting the systemic severity of the underlying disease. Pre-existing antithrombotic therapy was associated with a numerically higher stroke risk in this analysis, supporting discontinuation where no other indication exists. Improving stroke prevention in this population will require strategies other than antithrombotic therapy, and prospective studies and randomized trials are needed to identify these and to optimize acute treatment.

## Supplementary Information

Below is the link to the electronic supplementary material.

Supplementary Material 1

## Data Availability

The data that support the findings of this study are available from the corresponding author upon reasonable request.
