# Supplementary material for: Characterization and treatment of ischemic stroke in patients with infective endocarditis: a meta‐analysis
Source: Neurol Res Pract. 2026 Oct 26;8(1):89. doi: 10.1186/s42466-026-00542-z (PMC13712007; doi:10.1186/s42466-026-00542-z)
Supplement: Supplementary file 1 [file 42466_2026_542_MOESM1_ESM.pdf]

Characterization and treatment of ischemic stroke in patients with infective endocarditis: A meta-analysis

Supplementary Figures

Supplementary Figure 1 PRISMA flow diagram of the literature search and study selection. PRISMA, Preferred Reporting Items for Systematic reviews and Meta-Analyses[1]. PubMed was searched using the terms "*endocarditis*" AND ("*stroke*" OR "*ischemia*" OR "*infarction*") for articles published up to October 1, 2022. Inclusion criteria: adult patients (≥ 18 years), diagnosis of infective endocarditis, report on early stroke outcomes. Exclusion criteria: infective endocarditis associated with immunocompromised conditions such as HIV infection or solid organ or hematopoietic stem cell transplantation, infective endocarditis related to intravenous drug use or congenital heart disease, stroke occurring as complication of cardiac surgery, autopsy studies

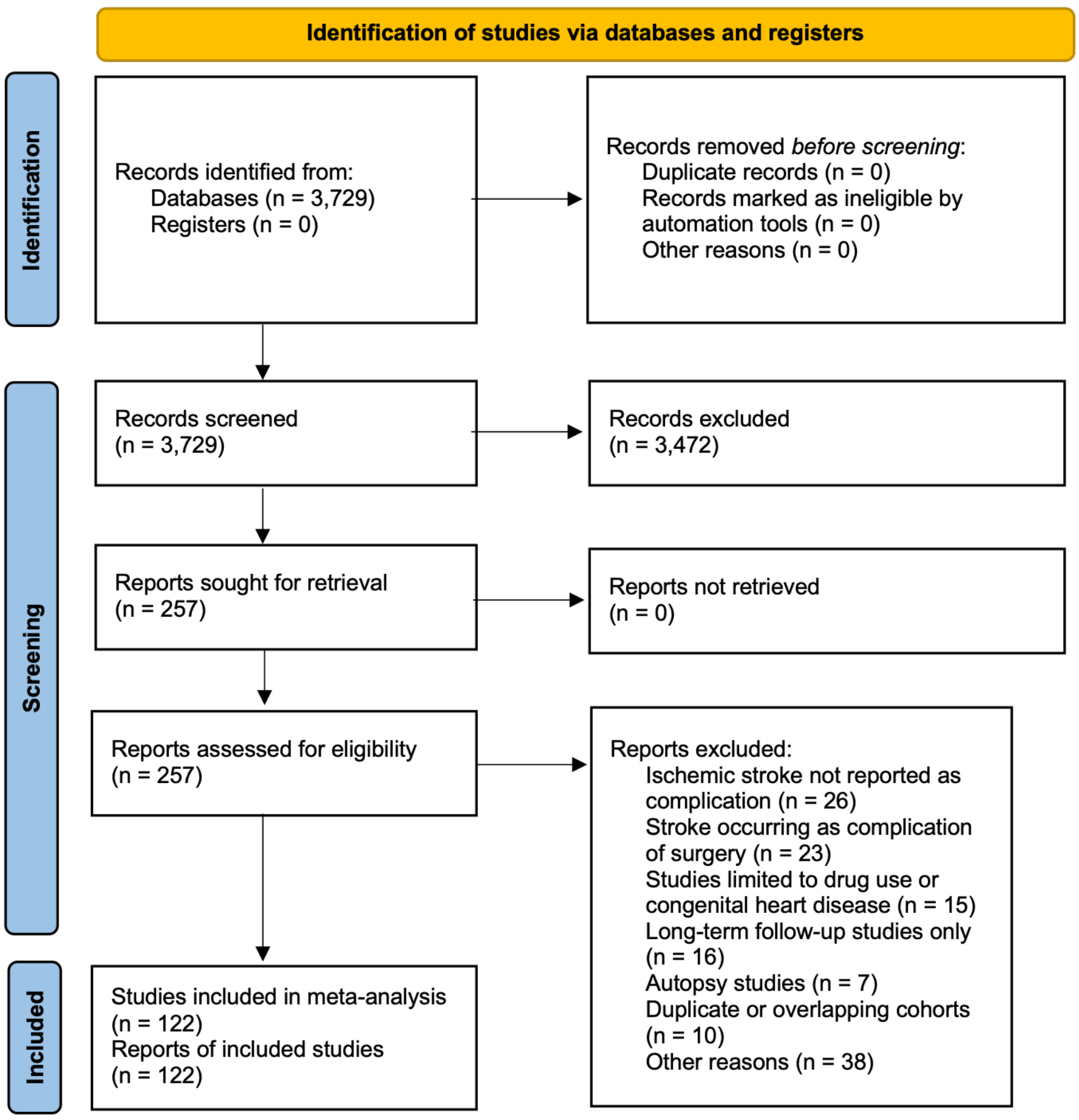

Supplementary Figure 2. Location of included studies

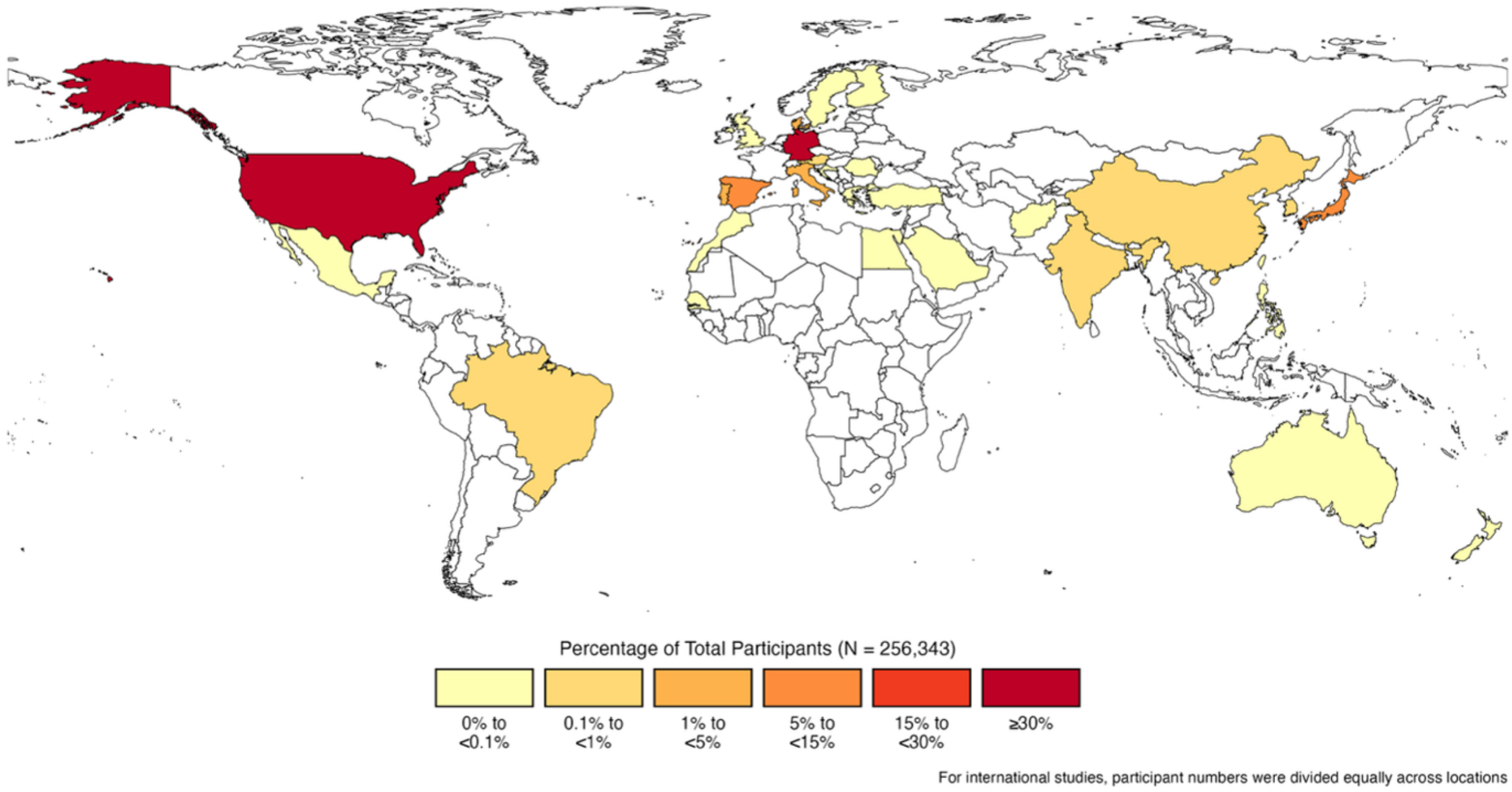

Supplementary Figure 3. Frequency of stroke in patients with infective endocarditis; A: Across all studies and patients, B: In patients with mitral valve endocarditis, C: In patients with aortic valve endocarditis, D: In patients with tricuspid valve endocarditis

A

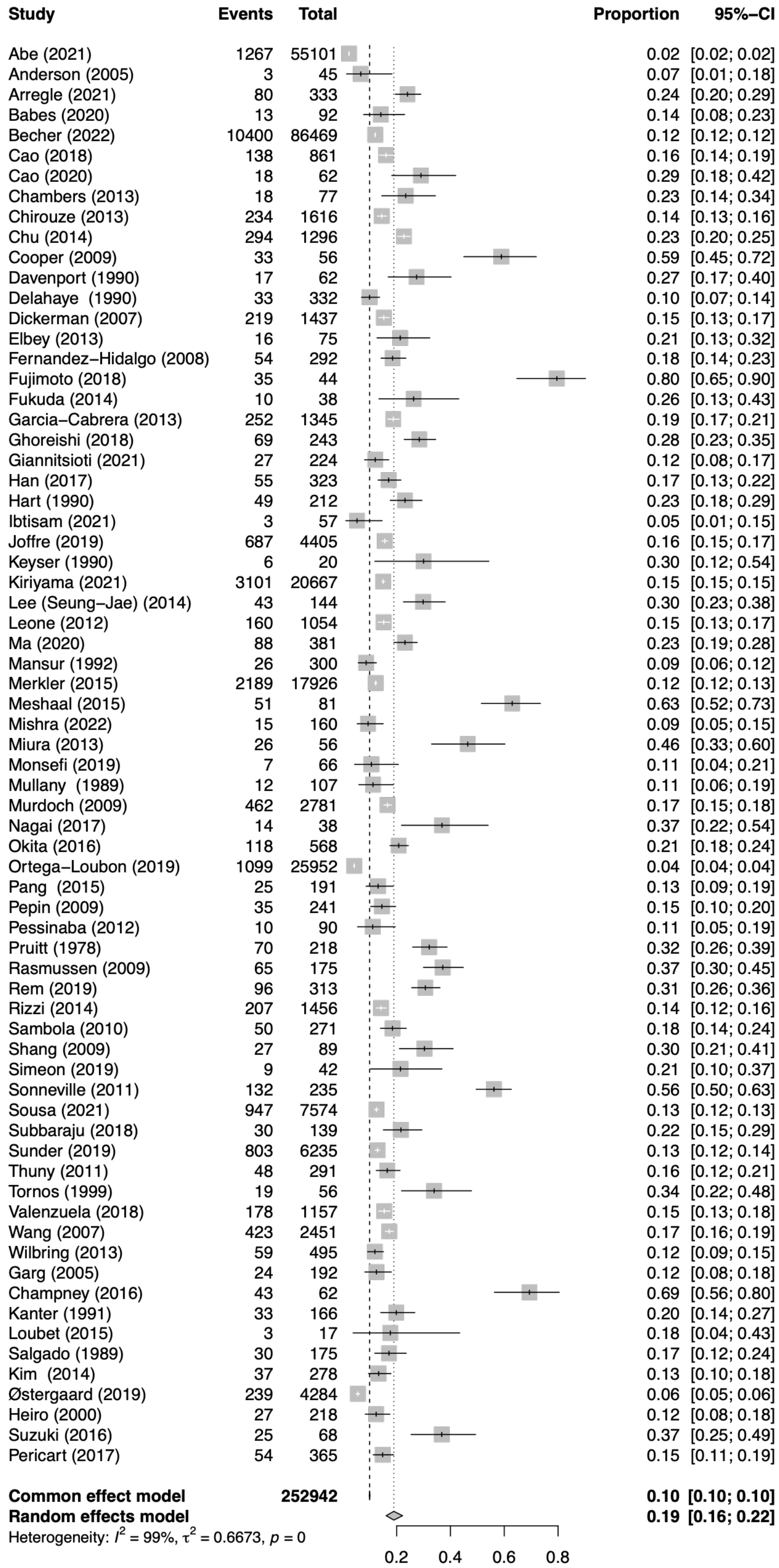

B

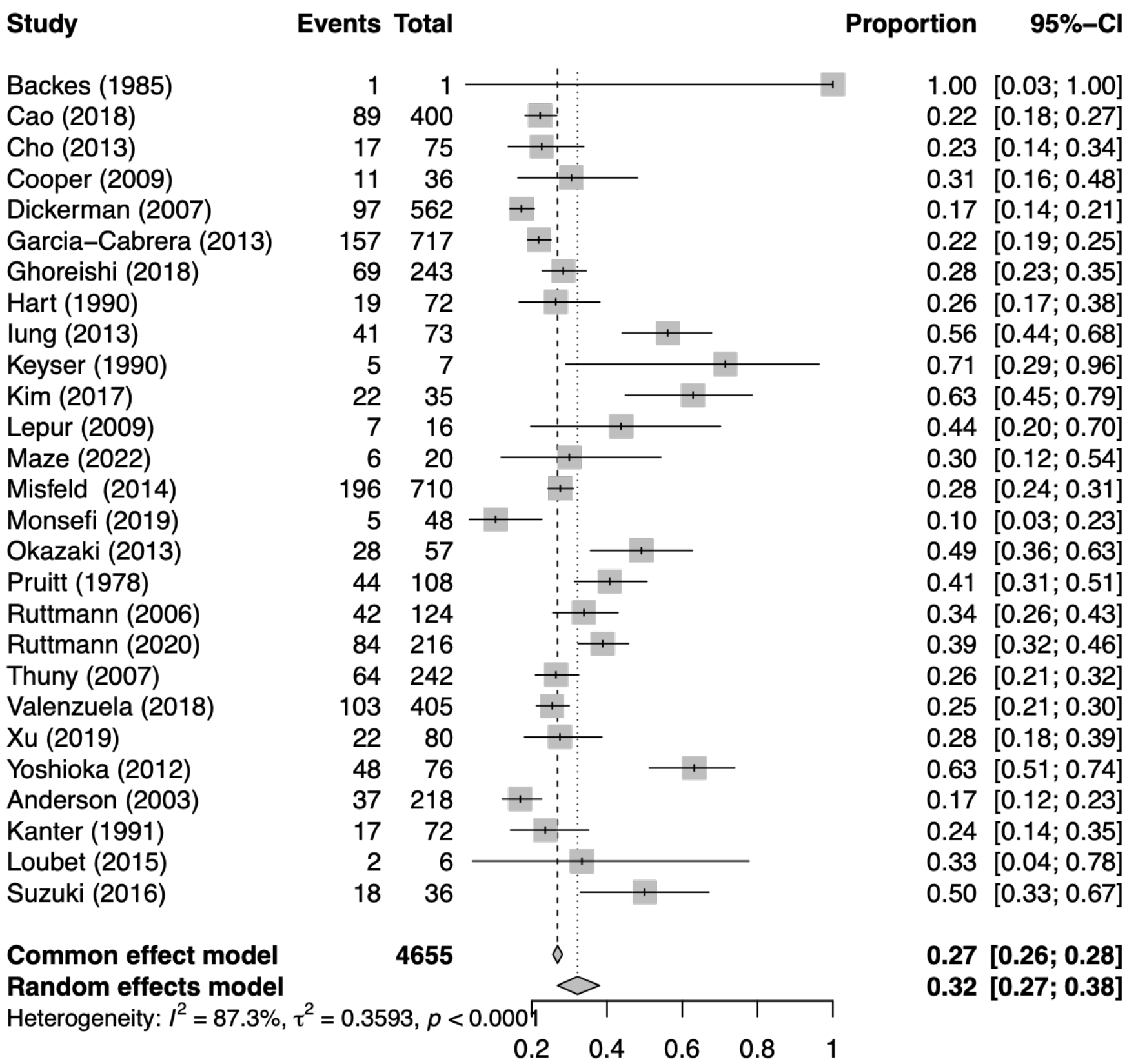
Study Events Total Proportion 95%−CI
Backes (1985) 1 1 1.00 [0.03; 1.00]
Cao (2018) 89 400 0.22 [0.18; 0.27]
Cho (2013) 17 75 0.23 [0.14; 0.34]
Cooper (2009) 11 36 0.31 [0.16; 0.48]
Dickerman (2007) 97 562 0.17 [0.14; 0.21]
Garcia−Cabrera (2013) 157 717 0.22 [0.19; 0.25]
Ghoreishi (2018) 69 243 0.28 [0.23; 0.35]
Hart (1990) 19 72 0.26 [0.17; 0.38]
Iung (2013) 41 73 0.56 [0.44; 0.68]
Keyser (1990) 5 7 0.71 [0.29; 0.96]
Kim (2017) 22 35 0.63 [0.45; 0.79]
Lepur (2009) 7 16 0.44 [0.20; 0.70]
Maze (2022) 6 20 0.30 [0.12; 0.54]
Misfeld (2014) 196 710 0.28 [0.24; 0.31]
Monsefi (2019) 5 48 0.10 [0.03; 0.23]
Okazaki (2013) 28 57 0.49 [0.36; 0.63]
Pruitt (1978) 44 108 0.41 [0.31; 0.51]
Ruttmann (2006) 42 124 0.34 [0.26; 0.43]
Ruttmann (2020) 84 216 0.39 [0.32; 0.46]
Thuny (2007) 64 242 0.26 [0.21; 0.32]
Valenzuela (2018) 103 405 0.25 [0.21; 0.30]
Xu (2019) 22 80 0.28 [0.18; 0.39]
Yoshioka (2012) 48 76 0.63 [0.51; 0.74]
Anderson (2003) 37 218 0.17 [0.12; 0.23]
Kanter (1991) 17 72 0.24 [0.14; 0.35]
Loubet (2015) 2 6 0.33 [0.04; 0.78]
Suzuki (2016) 18 36 0.50 [0.33; 0.67]
Common effect model 4655 0.27 [0.26; 0.28]
Random effects model 0.32 [0.27; 0.38]
Heterogeneity: I^2 = 87.3%, τ^2 = 0.3593, $p < 0.0001$
0.2 0.4 0.6 0.8 1

C

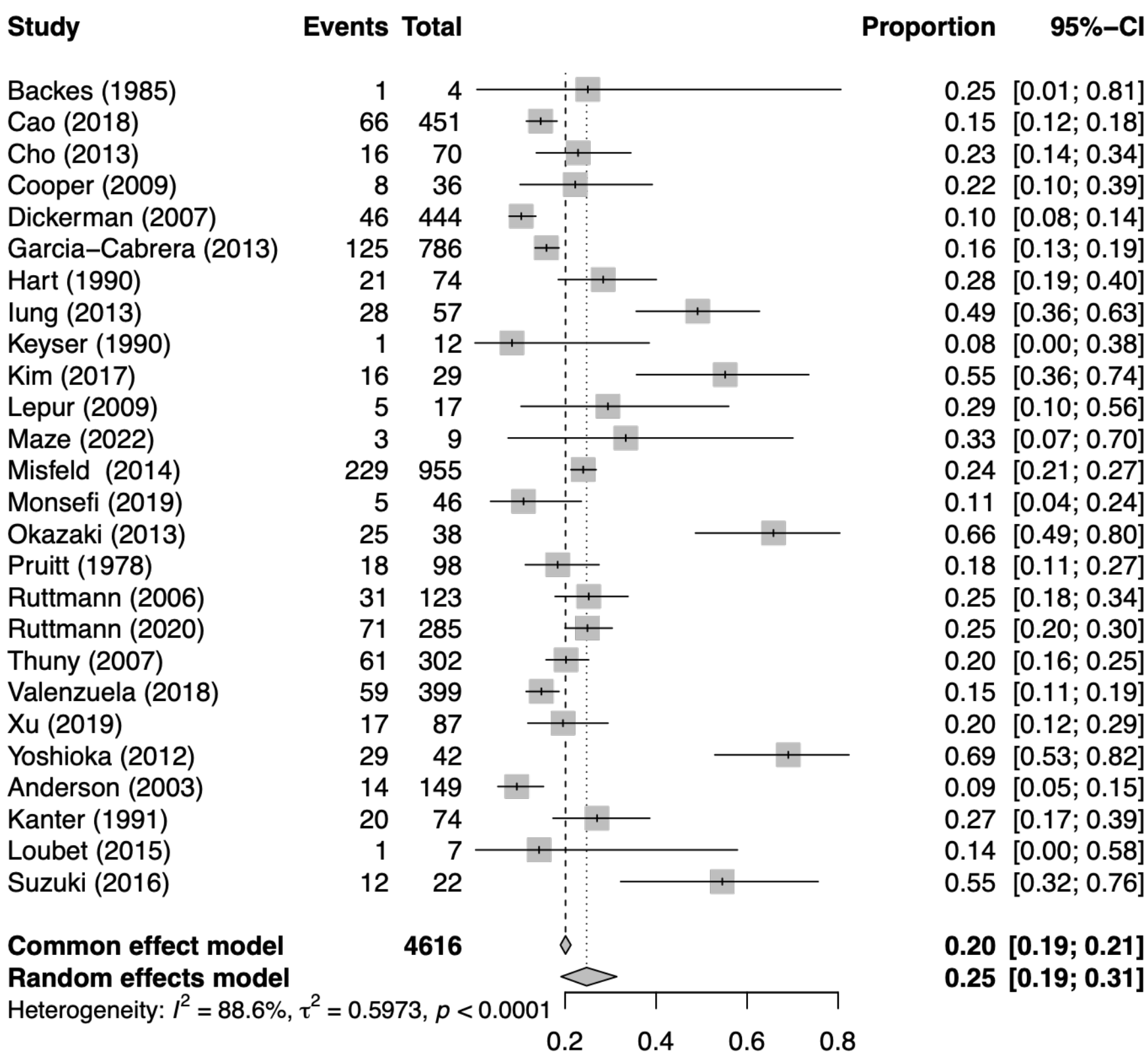
Study Events Total Proportion 95%−CI
Backes (1985) 1 4 0.25 [0.01; 0.81]
Cao (2018) 66 451 0.15 [0.12; 0.18]
Cho (2013) 16 70 0.23 [0.14; 0.34]
Cooper (2009) 8 36 0.22 [0.10; 0.39]
Dickerman (2007) 46 444 0.10 [0.08; 0.14]
Garcia−Cabrera (2013) 125 786 0.16 [0.13; 0.19]
Hart (1990) 21 74 0.28 [0.19; 0.40]
Iung (2013) 28 57 0.49 [0.36; 0.63]
Keyser (1990) 1 12 0.08 [0.00; 0.38]
Kim (2017) 16 29 0.55 [0.36; 0.74]
Lepur (2009) 5 17 0.29 [0.10; 0.56]
Maze (2022) 3 9 0.33 [0.07; 0.70]
Misfeld (2014) 229 955 0.24 [0.21; 0.27]
Monsefi (2019) 5 46 0.11 [0.04; 0.24]
Okazaki (2013) 25 38 0.66 [0.49; 0.80]
Pruitt (1978) 18 98 0.18 [0.11; 0.27]
Ruttmann (2006) 31 123 0.25 [0.18; 0.34]
Ruttmann (2020) 71 285 0.25 [0.20; 0.30]
Thuny (2007) 61 302 0.20 [0.16; 0.25]
Valenzuela (2018) 59 399 0.15 [0.11; 0.19]
Xu (2019) 17 87 0.20 [0.12; 0.29]
Yoshioka (2012) 29 42 0.69 [0.53; 0.82]
Anderson (2003) 14 149 0.09 [0.05; 0.15]
Kanter (1991) 20 74 0.27 [0.17; 0.39]
Loubet (2015) 1 7 0.14 [0.00; 0.58]
Suzuki (2016) 12 22 0.55 [0.32; 0.76]
Common effect model 4616 0.20 [0.19; 0.21]
Random effects model 0.25 [0.19; 0.31]
Heterogeneity: I^2 = 88.6%, τ^2 = 0.5973, $p < 0.0001$
0.2 0.4 0.6 0.8

D

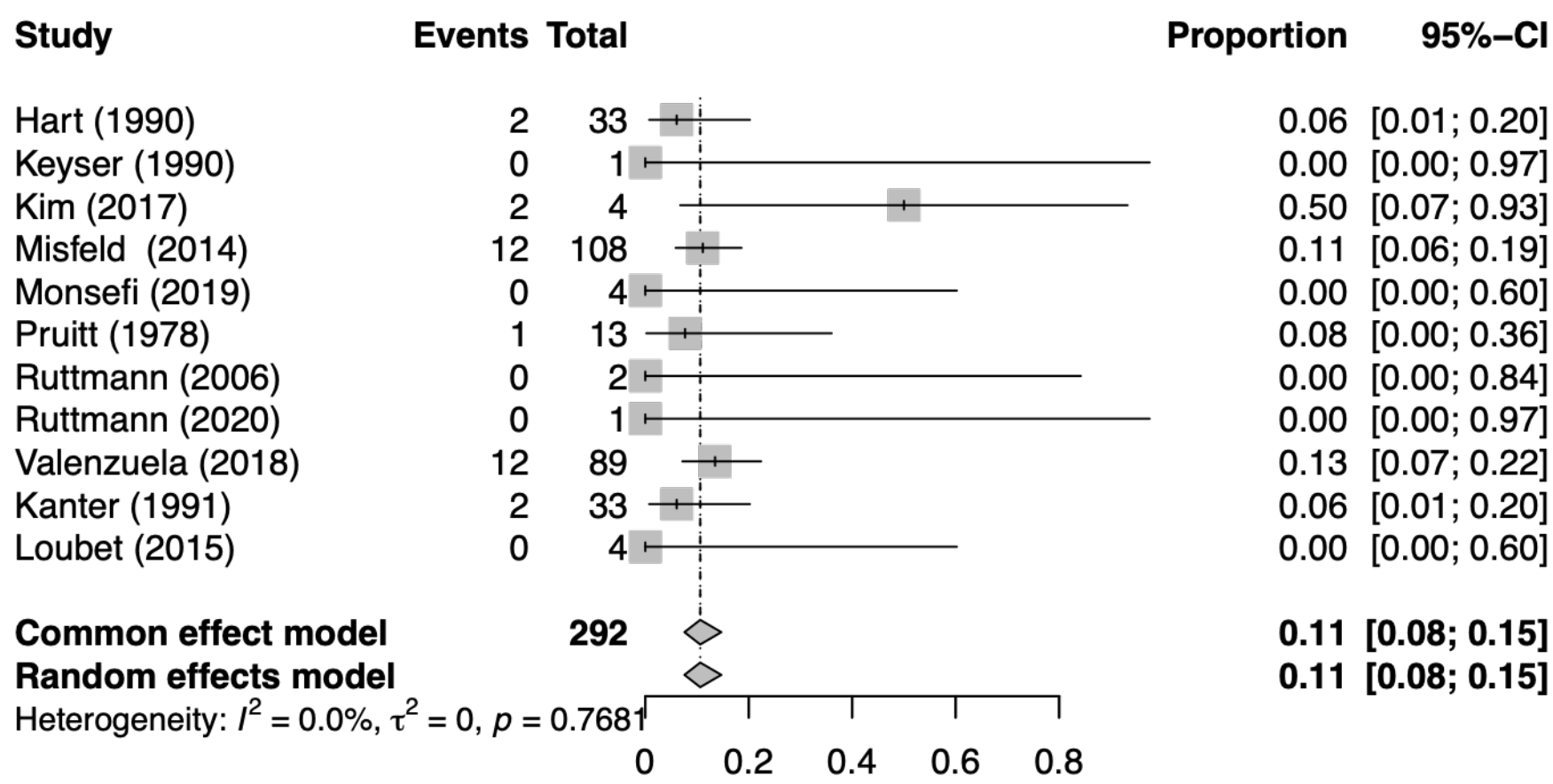
Study Events Total Proportion 95%−CI
Hart (1990) 2 33 0.06 [0.01; 0.20]
Keyser (1990) 0 1 0.00 [0.00; 0.97]
Kim (2017) 2 4 0.50 [0.07; 0.93]
Misfeld (2014) 12 108 0.11 [0.06; 0.19]
Monsefi (2019) 0 4 0.00 [0.00; 0.60]
Pruitt (1978) 1 13 0.08 [0.00; 0.36]
Ruttmann (2006) 0 2 0.00 [0.00; 0.84]
Ruttmann (2020) 0 1 0.00 [0.00; 0.97]
Valenzuela (2018) 12 89 0.13 [0.07; 0.22]
Kanter (1991) 2 33 0.06 [0.01; 0.20]
Loubet (2015) 0 4 0.00 [0.00; 0.60]
Common effect model 292 0.11 [0.08; 0.15]
Random effects model 0.11 [0.08; 0.15]
Heterogeneity: I^2 = 0.0%, τ^2 = 0, p = 0.7681
0 0.2 0.4 0.6 0.8

Supplementary Figure 4. Frequency of stroke in patients with A enterococcal, B streptococcal, C staphylococcal, D others and E blood culture negative infective endocarditis

A

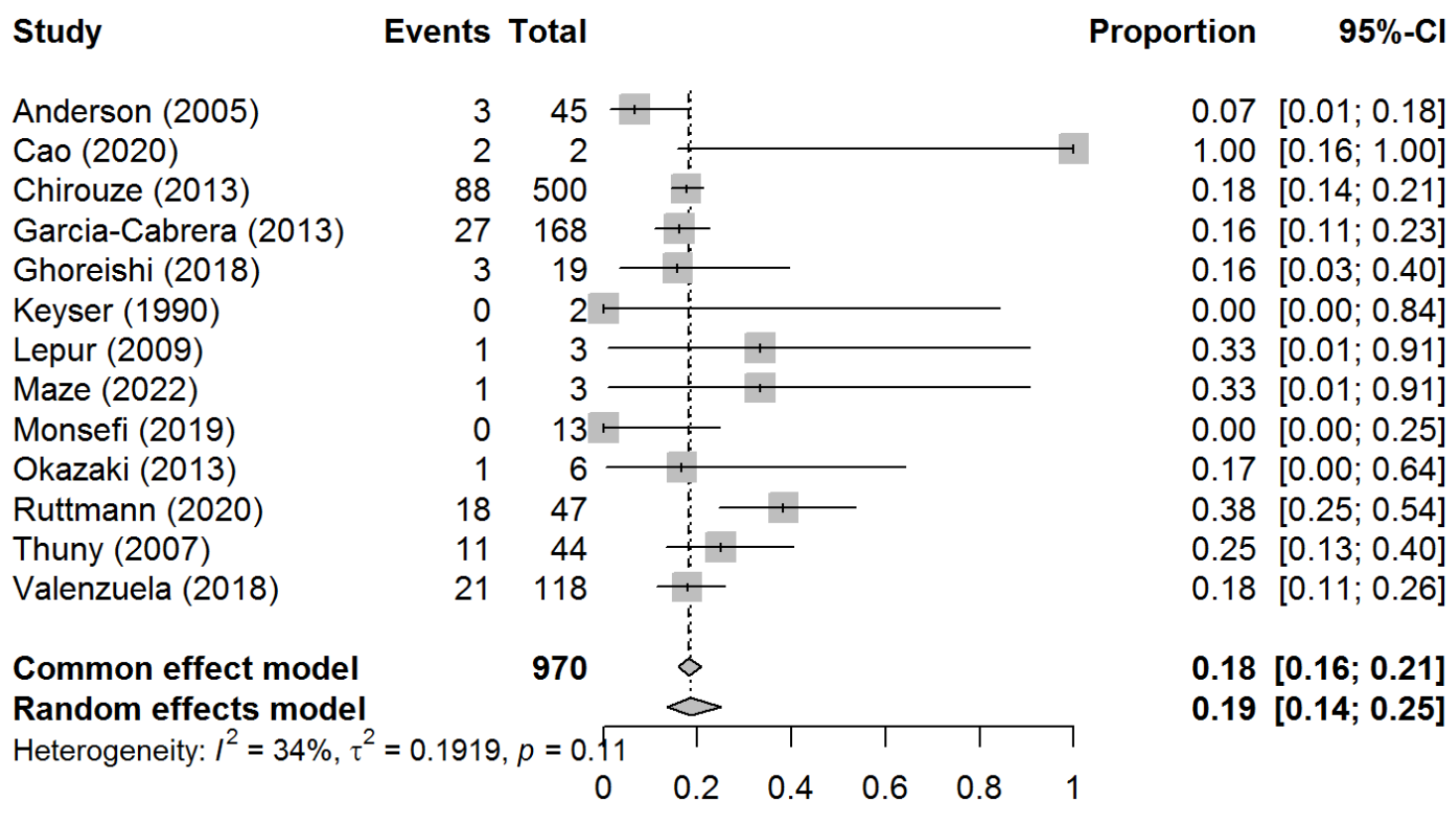

B

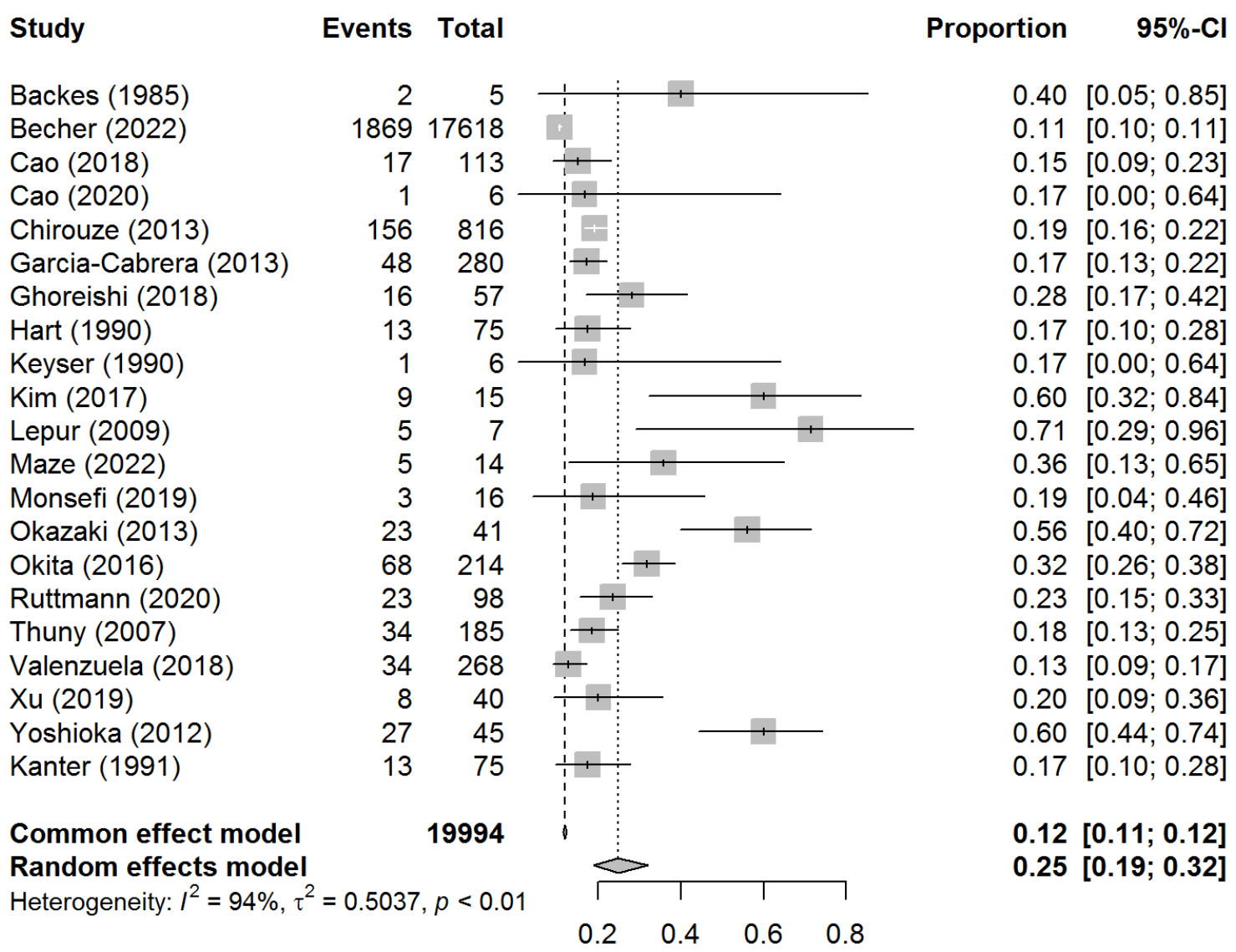

C

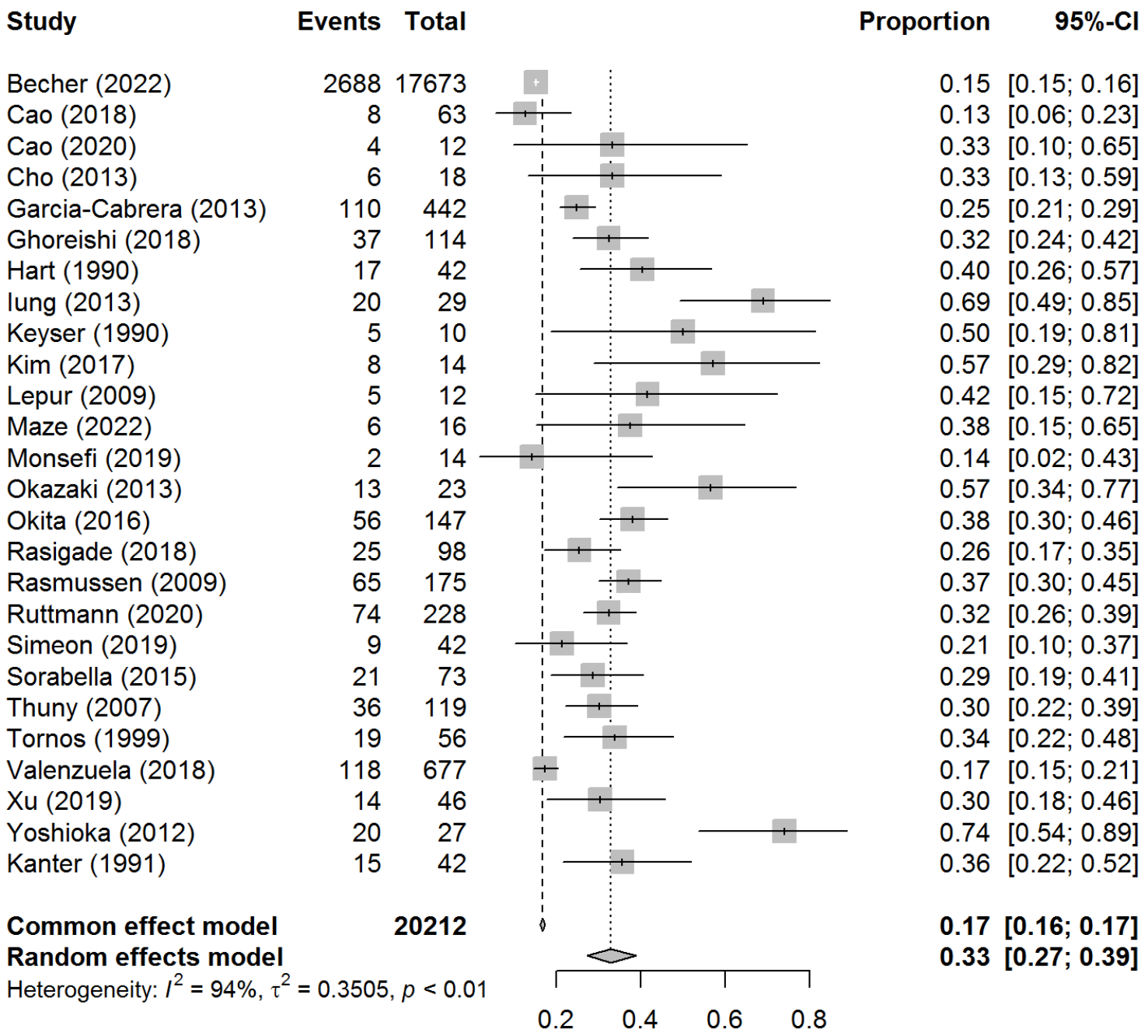
Study Events Total Proportion 95%-CI
Becher (2022) 2688 17673 0.15 [0.15; 0.16]
Cao (2018) 8 63 0.13 [0.06; 0.23]
Cao (2020) 4 12 0.33 [0.10; 0.65]
Cho (2013) 6 18 0.33 [0.13; 0.59]
Garcia-Cabrera (2013) 110 442 0.25 [0.21; 0.29]
Ghoreishi (2018) 37 114 0.32 [0.24; 0.42]
Hart (1990) 17 42 0.40 [0.26; 0.57]
Iung (2013) 20 29 0.69 [0.49; 0.85]
Keyser (1990) 5 10 0.50 [0.19; 0.81]
Kim (2017) 8 14 0.57 [0.29; 0.82]
Lepur (2009) 5 12 0.42 [0.15; 0.72]
Maze (2022) 6 16 0.38 [0.15; 0.65]
Monsefi (2019) 2 14 0.14 [0.02; 0.43]
Okazaki (2013) 13 23 0.57 [0.34; 0.77]
Okita (2016) 56 147 0.38 [0.30; 0.46]
Rasigade (2018) 25 98 0.26 [0.17; 0.35]
Rasmussen (2009) 65 175 0.37 [0.30; 0.45]
Ruttmann (2020) 74 228 0.32 [0.26; 0.39]
Simeon (2019) 9 42 0.21 [0.10; 0.37]
Sorabella (2015) 21 73 0.29 [0.19; 0.41]
Thuny (2007) 36 119 0.30 [0.22; 0.39]
Tornos (1999) 19 56 0.34 [0.22; 0.48]
Valenzuela (2018) 118 677 0.17 [0.15; 0.21]
Xu (2019) 14 46 0.30 [0.18; 0.46]
Yoshioka (2012) 20 27 0.74 [0.54; 0.89]
Kanter (1991) 15 42 0.36 [0.22; 0.52]
Common effect model 20212 0.17 [0.16; 0.17]
Random effects model 0.33 [0.27; 0.39]
Heterogeneity: I^2 = 94%, τ^2 = 0.3505, p < 0.01
0.2 0.4 0.6 0.8

D

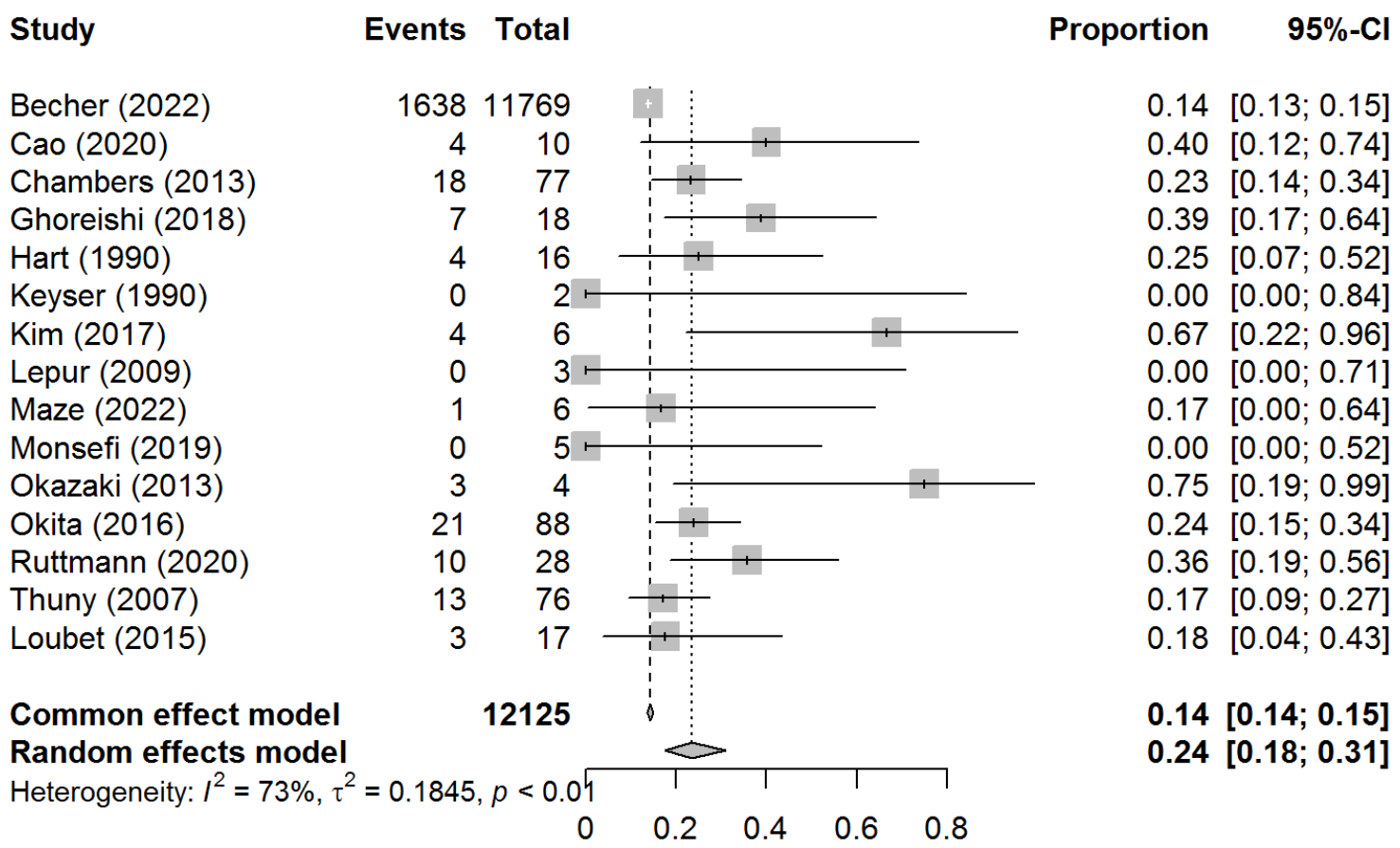
Study Events Total Proportion 95%-CI
Becher (2022) 1638 11769 0.14 [0.13; 0.15]
Cao (2020) 4 10 0.40 [0.12; 0.74]
Chambers (2013) 18 77 0.23 [0.14; 0.34]
Ghoreishi (2018) 7 18 0.39 [0.17; 0.64]
Hart (1990) 4 16 0.25 [0.07; 0.52]
Keyser (1990) 0 2 0.00 [0.00; 0.84]
Kim (2017) 4 6 0.67 [0.22; 0.96]
Lepur (2009) 0 3 0.00 [0.00; 0.71]
Maze (2022) 1 6 0.17 [0.00; 0.64]
Monsefi (2019) 0 5 0.00 [0.00; 0.52]
Okazaki (2013) 3 4 0.75 [0.19; 0.99]
Okita (2016) 21 88 0.24 [0.15; 0.34]
Ruttmann (2020) 10 28 0.36 [0.19; 0.56]
Thuny (2007) 13 76 0.17 [0.09; 0.27]
Loubet (2015) 3 17 0.18 [0.04; 0.43]
Common effect model 12125 0.14 [0.14; 0.15]
Random effects model 0.24 [0.18; 0.31]
Heterogeneity: I^2 = 73%, τ^2 = 0.1845, p < 0.01
0 0.2 0.4 0.6 0.8

E

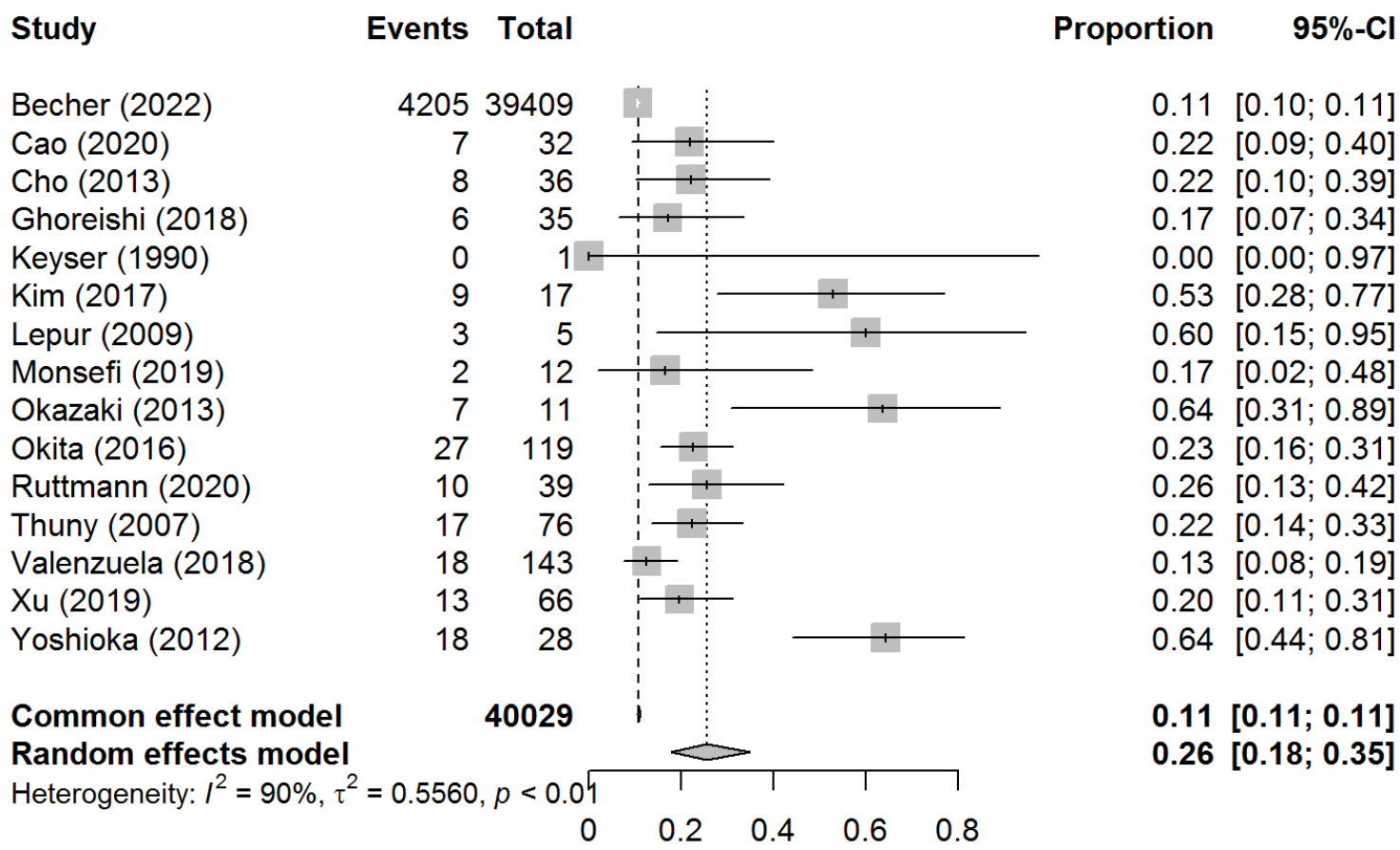

Study
Events
Total
Proportion
95%-CI
Becher (2022) 4205 39409 0.11 [0.10; 0.11]
Cao (2020) 7 32 0.22 [0.09; 0.40]
Cho (2013) 8 36 0.22 [0.10; 0.39]
Ghoreishi (2018) 6 35 0.17 [0.07; 0.34]
Keyser (1990) 0 1 0.00 [0.00; 0.97]
Kim (2017) 9 17 0.53 [0.28; 0.77]
Lepur (2009) 3 5 0.60 [0.15; 0.95]
Monsefi (2019) 2 12 0.17 [0.02; 0.48]
Okazaki (2013) 7 11 0.64 [0.31; 0.89]
Okita (2016) 27 119 0.23 [0.16; 0.31]
Ruttmann (2020) 10 39 0.26 [0.13; 0.42]
Thuny (2007) 17 76 0.22 [0.14; 0.33]
Valenzuela (2018) 18 143 0.13 [0.08; 0.19]
Xu (2019) 13 66 0.20 [0.11; 0.31]
Yoshioka (2012) 18 28 0.64 [0.44; 0.81]
Common effect model 40029 0.11 [0.11; 0.11]
Random effects model 0.26 [0.18; 0.35]
Heterogeneity: I^2 = 90%, τ^2 = 0.5560, p < 0.01
0
0.2
0.4
0.6
0.8

Supplementary Figure 5. Frequency of A enterococcal, B streptococcal, C staphylococcal, D others and E blood culture negative endocarditis in patients with stroke due to infective endocarditis

A

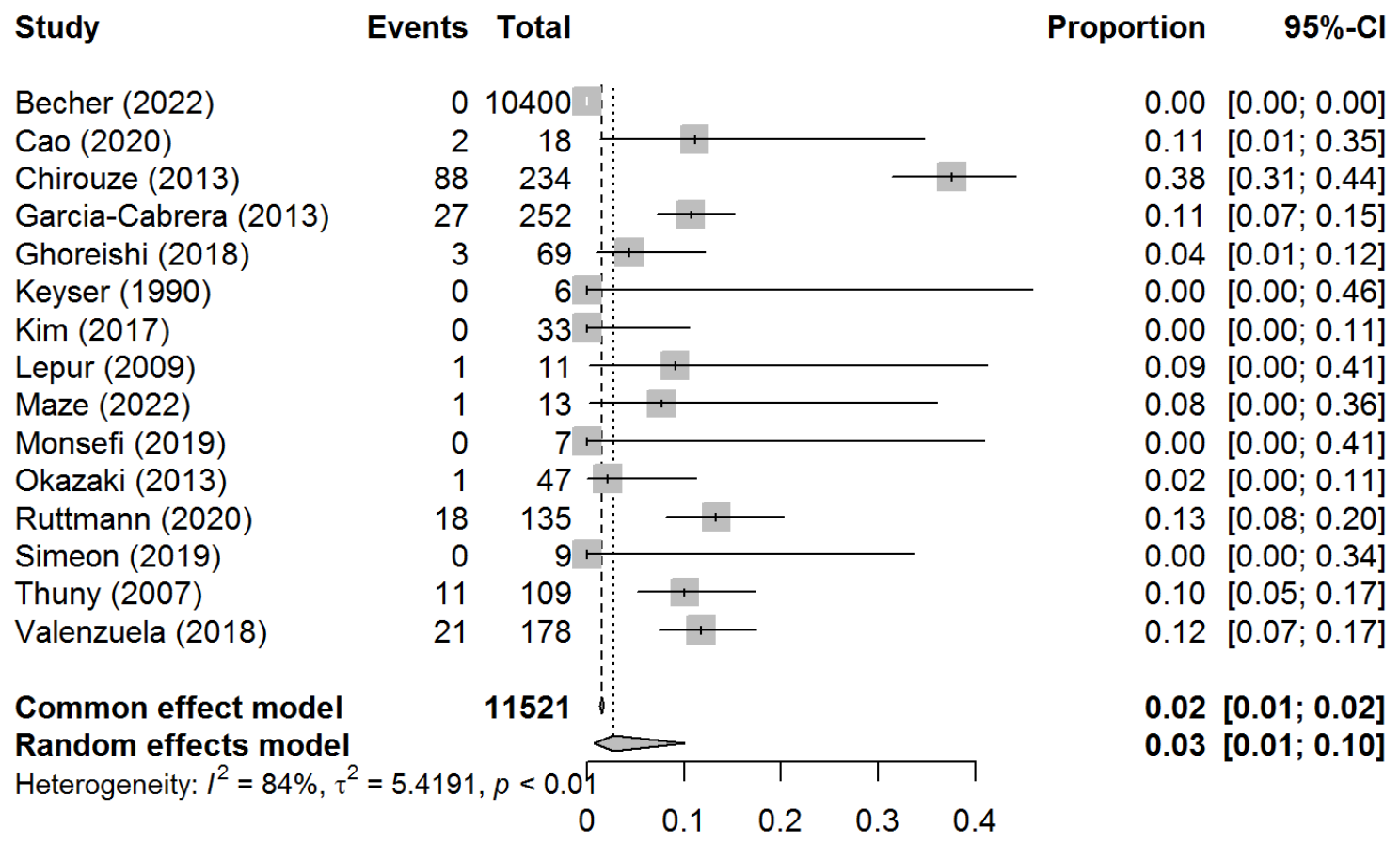

B

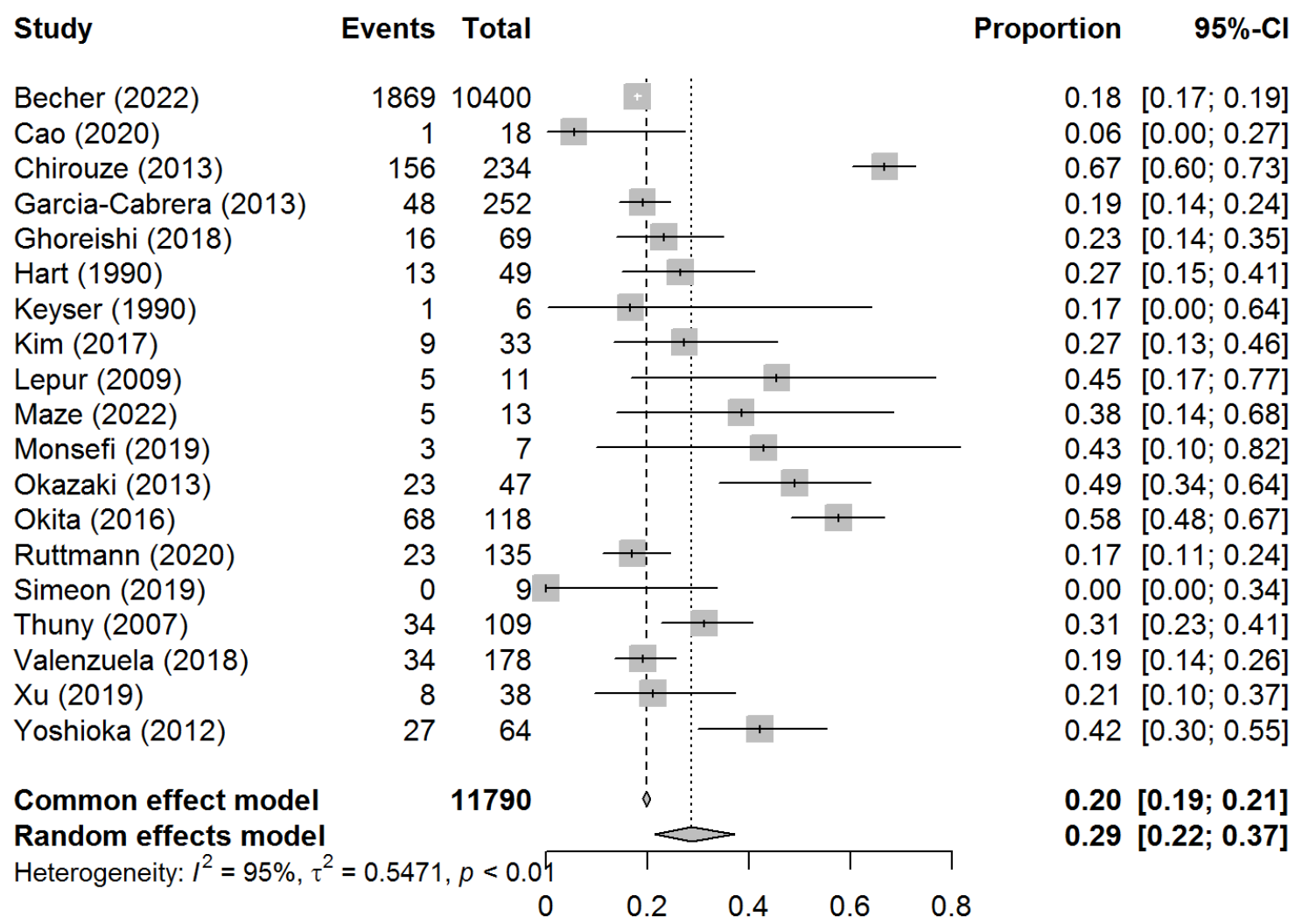

C

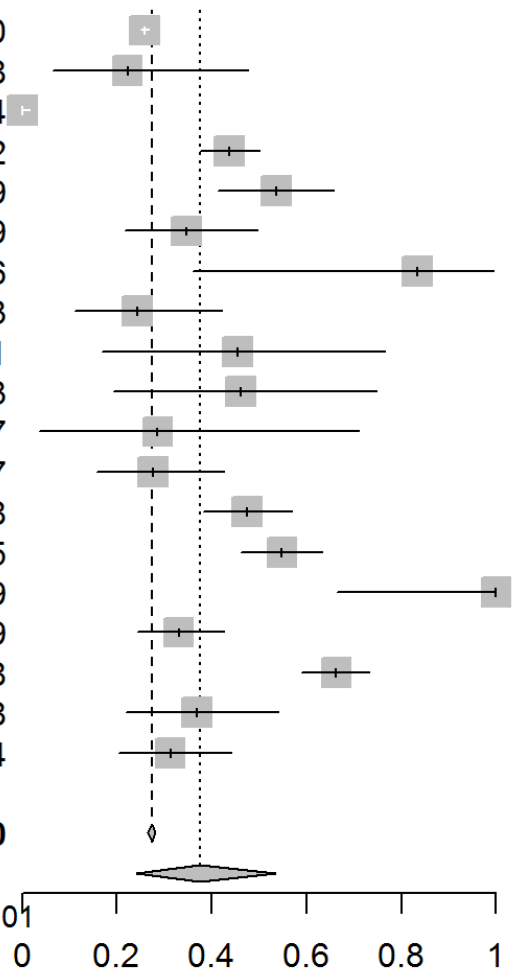

Study	Events	Total	Proportion	95%-CI
Becher (2022)	2688	10400	0.26	[0.25; 0.27]
Cao (2020)	4	18	0.22	[0.06; 0.48]
Chirouze (2013)	0	234	0.00	[0.00; 0.02]
Garcia-Cabrera (2013)	110	252	0.44	[0.37; 0.50]
Ghoreishi (2018)	37	69	0.54	[0.41; 0.66]
Hart (1990)	17	49	0.35	[0.22; 0.50]
Keyser (1990)	5	6	0.83	[0.36; 1.00]
Kim (2017)	8	33	0.24	[0.11; 0.42]
Lepur (2009)	5	11	0.45	[0.17; 0.77]
Maze (2022)	6	13	0.46	[0.19; 0.75]
Monsefi (2019)	2	7	0.29	[0.04; 0.71]
Okazaki (2013)	13	47	0.28	[0.16; 0.43]
Okita (2016)	56	118	0.47	[0.38; 0.57]
Ruttmann (2020)	74	135	0.55	[0.46; 0.63]
Simeon (2019)	9	9	1.00	[0.66; 1.00]
Thuny (2007)	36	109	0.33	[0.24; 0.43]
Valenzuela (2018)	118	178	0.66	[0.59; 0.73]
Xu (2019)	14	38	0.37	[0.22; 0.54]
Yoshioka (2012)	20	64	0.31	[0.20; 0.44]
**Common effect model**		**11790**	**0.27**	**[0.27; 0.28]**
**Random effects model**			**0.38**	**[0.24; 0.53]**

Heterogeneity: $I^2 = 93\%$, $\tau^2 = 1.7971$, $p < 0.01$

D

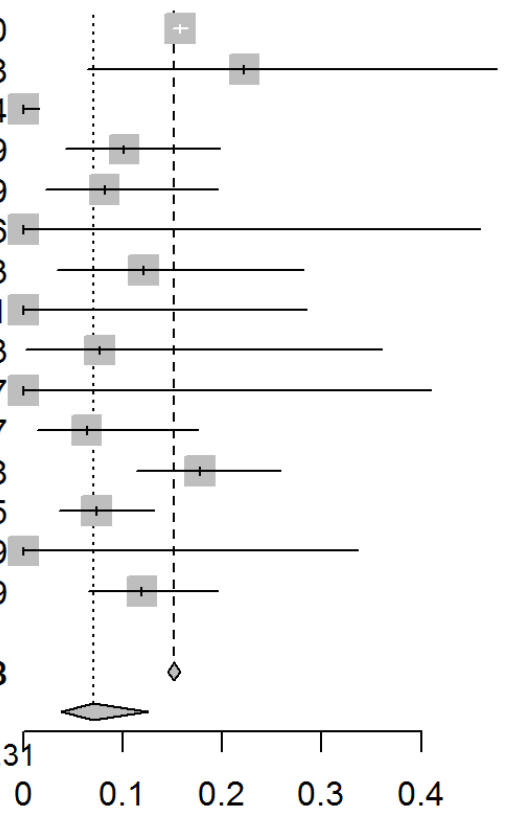

Study	Events	Total	Proportion	95%-CI
Becher (2022)	1638	10400	0.16	[0.15; 0.16]
Cao (2020)	4	18	0.22	[0.06; 0.48]
Chirouze (2013)	0	234	0.00	[0.00; 0.02]
Ghoreishi (2018)	7	69	0.10	[0.04; 0.20]
Hart (1990)	4	49	0.08	[0.02; 0.20]
Keyser (1990)	0	6	0.00	[0.00; 0.46]
Kim (2017)	4	33	0.12	[0.03; 0.28]
Lepur (2009)	0	11	0.00	[0.00; 0.28]
Maze (2022)	1	13	0.08	[0.00; 0.36]
Monsefi (2019)	0	7	0.00	[0.00; 0.41]
Okazaki (2013)	3	47	0.06	[0.01; 0.18]
Okita (2016)	21	118	0.18	[0.11; 0.26]
Ruttmann (2020)	10	135	0.07	[0.04; 0.13]
Simeon (2019)	0	9	0.00	[0.00; 0.34]
Thuny (2007)	13	109	0.12	[0.07; 0.20]
**Common effect model**		**11258**	**0.15**	**[0.14; 0.16]**
**Random effects model**			**0.07**	**[0.04; 0.12]**

Heterogeneity: $I^2 = 12\%$, $\tau^2 = 0.8587$, $p = 0.31$

E

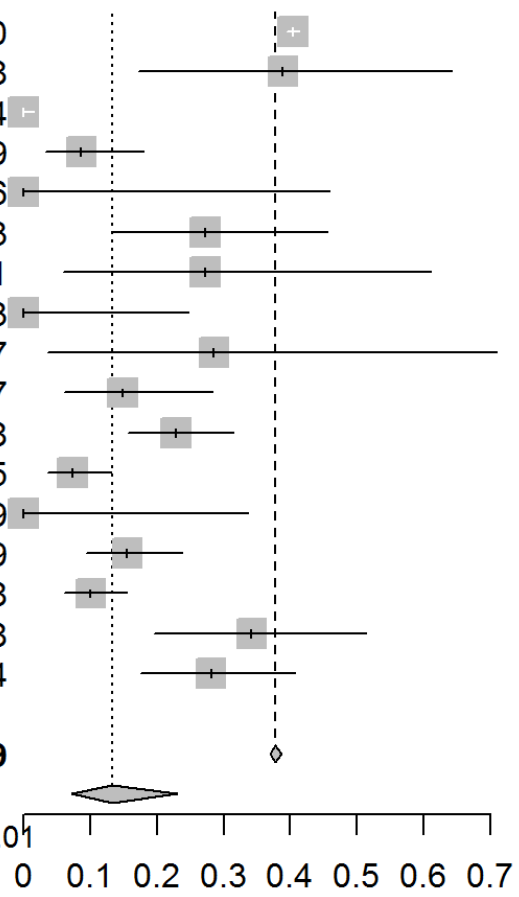

Study	Events	Total	Proportion	95%-CI
Becher (2022)	4205	10400	0.40	[0.39; 0.41]
Cao (2020)	7	18	0.39	[0.17; 0.64]
Chirouze (2013)	0	234	0.00	[0.00; 0.02]
Ghoreishi (2018)	6	69	0.09	[0.03; 0.18]
Keyser (1990)	0	6	0.00	[0.00; 0.46]
Kim (2017)	9	33	0.27	[0.13; 0.46]
Lepur (2009)	3	11	0.27	[0.06; 0.61]
Maze (2022)	0	13	0.00	[0.00; 0.25]
Monsefi (2019)	2	7	0.29	[0.04; 0.71]
Okazaki (2013)	7	47	0.15	[0.06; 0.28]
Okita (2016)	27	118	0.23	[0.16; 0.32]
Ruttmann (2020)	10	135	0.07	[0.04; 0.13]
Simeon (2019)	0	9	0.00	[0.00; 0.34]
Thuny (2007)	17	109	0.16	[0.09; 0.24]
Valenzuela (2018)	18	178	0.10	[0.06; 0.16]
Xu (2019)	13	38	0.34	[0.20; 0.51]
Yoshioka (2012)	18	64	0.28	[0.18; 0.41]
**Common effect model**		**11489**	**0.38**	**[0.37; 0.39]**
**Random effects model**			**0.13**	**[0.07; 0.23]**

Heterogeneity: $I^2 = 90\%$, $\tau^2 = 1.4954$, $p < 0.01$

Supplementary Figure 6. Frequency of stroke in patients with IE A with pre-existing anticoagulation or antiplatelet therapy and B without prior anticoagulation or antiplatelet therapy. Frequency of pre-existing anticoagulation or antiplatelet therapy in patients with IE C with stroke and D without stroke

A

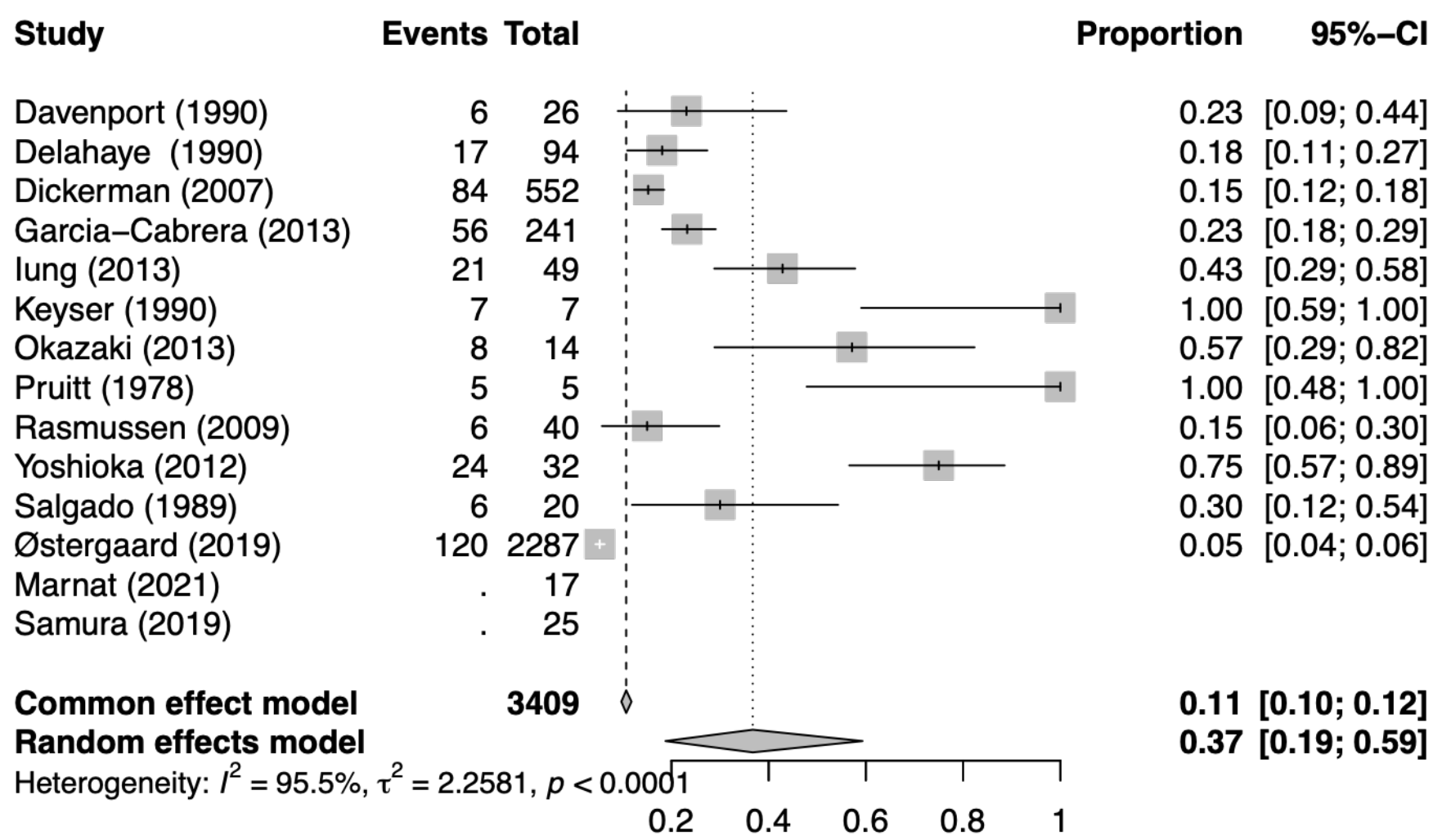

B

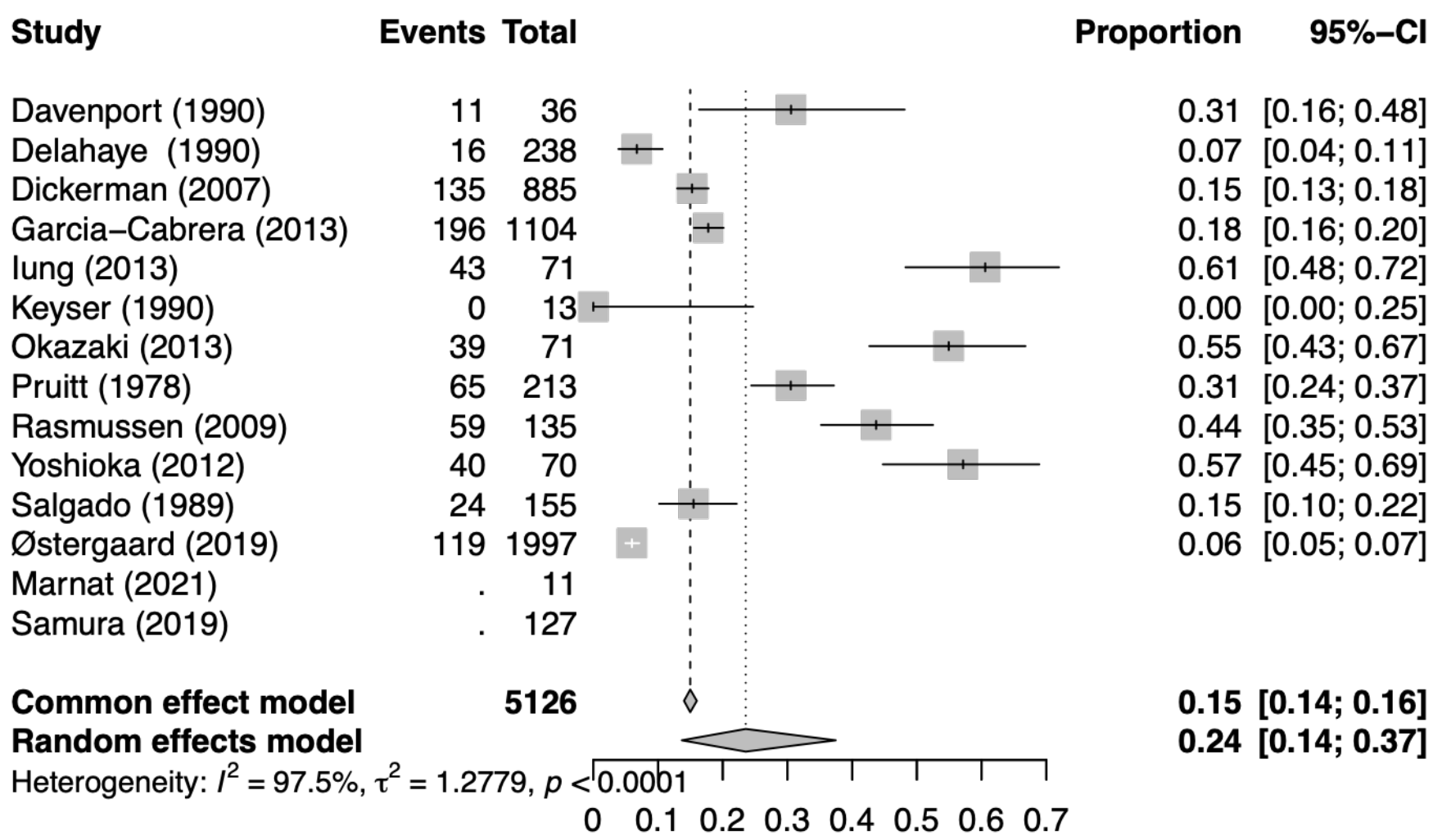

C

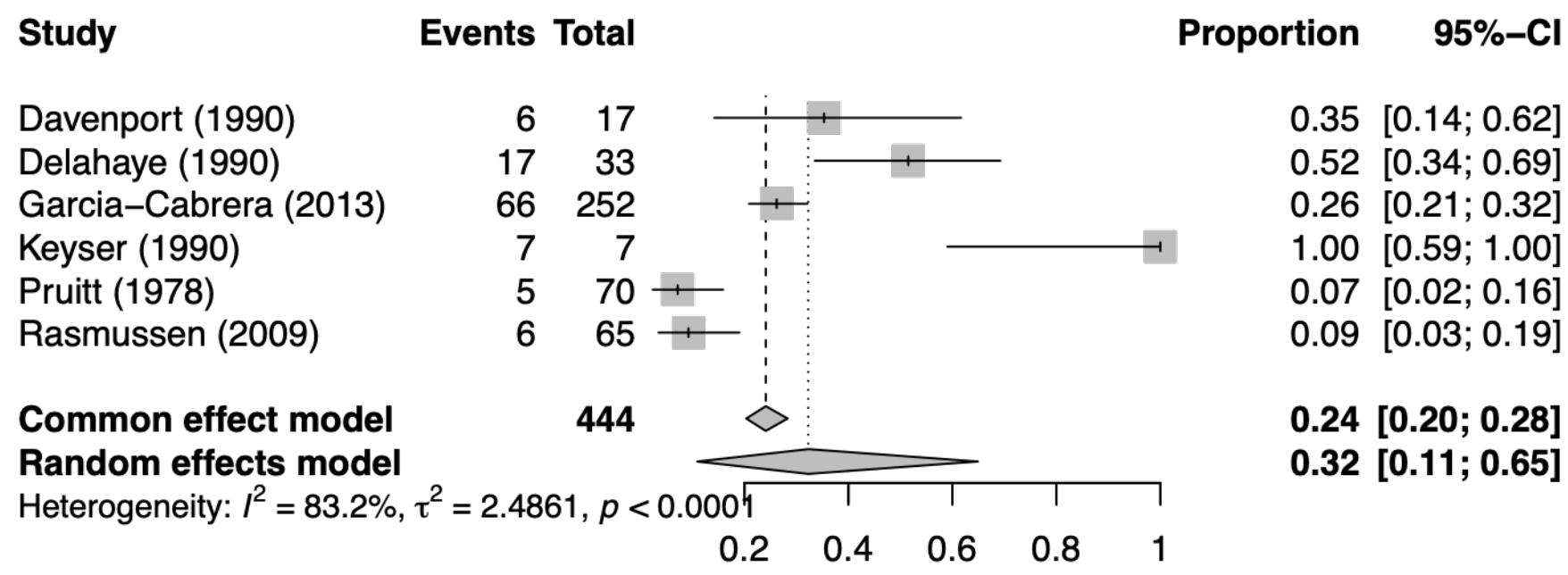
Study Events Total Proportion 95%−CI
Davenport (1990) 6 17 0.35 [0.14; 0.62]
Delahaye (1990) 17 33 0.52 [0.34; 0.69]
Garcia−Cabrera (2013) 66 252 0.26 [0.21; 0.32]
Keyser (1990) 7 7 1.00 [0.59; 1.00]
Pruitt (1978) 5 70 0.07 [0.02; 0.16]
Rasmussen (2009) 6 65 0.09 [0.03; 0.19]
Common effect model 444 0.24 [0.20; 0.28]
Random effects model 0.32 [0.11; 0.65]
Heterogeneity: I2 = 83.2%, τ2 = 2.4861, p < 0.0001
0.2 0.4 0.6 0.8 1

D

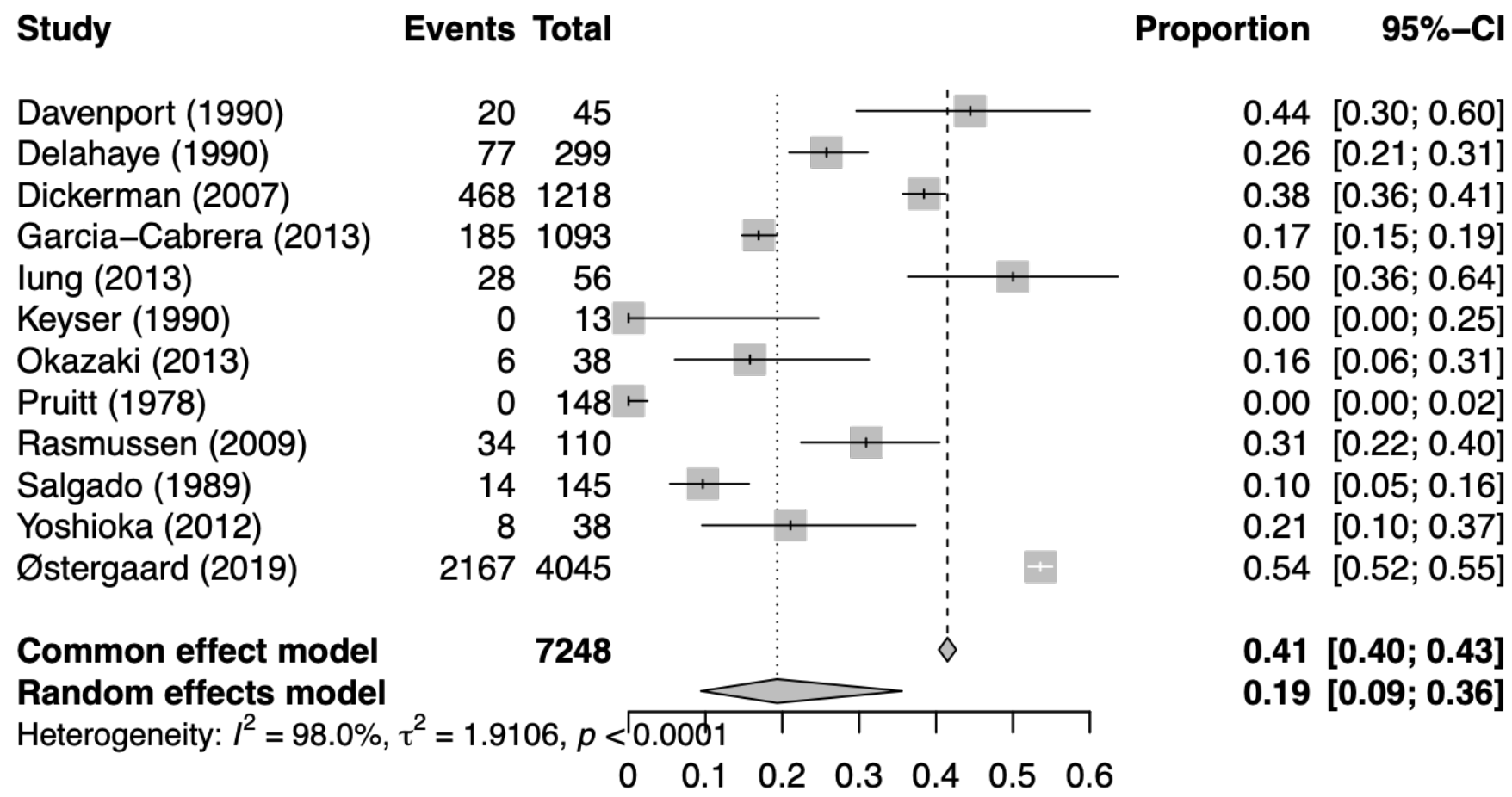
Study Events Total Proportion 95%−CI
Davenport (1990) 20 45 0.44 [0.30; 0.60]
Delahaye (1990) 77 299 0.26 [0.21; 0.31]
Dickerman (2007) 468 1218 0.38 [0.36; 0.41]
Garcia−Cabrera (2013) 185 1093 0.17 [0.15; 0.19]
Iung (2013) 28 56 0.50 [0.36; 0.64]
Keyser (1990) 0 13 0.00 [0.00; 0.25]
Okazaki (2013) 6 38 0.16 [0.06; 0.31]
Pruitt (1978) 0 148 0.00 [0.00; 0.02]
Rasmussen (2009) 34 110 0.31 [0.22; 0.40]
Salgado (1989) 14 145 0.10 [0.05; 0.16]
Yoshioka (2012) 8 38 0.21 [0.10; 0.37]
Østergaard (2019) 2167 4045 0.54 [0.52; 0.55]
Common effect model 7248 0.41 [0.40; 0.43]
Random effects model 0.19 [0.09; 0.36]
Heterogeneity: I2 = 98.0%, τ2 = 1.9106, p < 0.0001
0 0.1 0.2 0.3 0.4 0.5 0.6

Supplementary Figure 7. Mortality in patients with stroke due to infective endocarditis

Study	Stroke Events	Stroke Total	No Stroke Events	No Stroke Total	OR	95%-CI	Weight (common)	Weight (random)
Arregle (2021)	20	80	39	253	1.83	[0.99; 3.37]	4.1%	4.5%
Backes (1985)	2	2	0	3	35.00	[0.50; 2435.69]	0.0%	0.5%
Cao (2018)	17	138	13	723	7.67	[3.63; 16.20]	1.1%	4.1%
Cao (2020)	5	18	3	44	5.26	[1.10; 25.05]	0.4%	2.2%
Cho (2013)	4	26	8	85	1.75	[0.48; 6.36]	0.9%	2.7%
Cooper (2009)	6	33	9	23	0.35	[0.10; 1.17]	2.5%	2.9%
Elbey (2013)	14	16	12	59	27.42	[5.47; 137.37]	0.2%	2.1%
Fukuda (2014)	1	10	1	28	3.00	[0.17; 53.05]	0.1%	0.9%
Garcia-Cabrera (2013)	110	252	291	1093	2.13	[1.61; 2.83]	17.9%	5.3%
Giannitsioti (2021)	11	27	40	197	2.70	[1.16; 6.27]	1.7%	3.8%
Keyser (1990)	3	6	7	14	1.00	[0.15; 6.77]	0.6%	1.7%
Kim (2017)	5	33	1	22	3.75	[0.41; 34.54]	0.3%	1.4%
Leone (2012)	52	160	101	894	3.78	[2.56; 5.59]	6.0%	5.1%
Lepur (2009)	4	11	3	19	3.05	[0.53; 17.37]	0.4%	1.9%
Ma (2020)	17	88	15	293	4.44	[2.11; 9.32]	1.6%	4.1%
Maze (2022)	3	13	3	26	2.30	[0.39; 13.42]	0.4%	1.9%
Misfeld (2014)	81	375	235	1196	1.13	[0.85; 1.50]	25.6%	5.3%
Monsefi (2019)	0	7	11	59	0.28	[0.01; 5.29]	0.7%	0.9%
Nagai (2017)	2	14	8	24	0.33	[0.06; 1.86]	1.5%	1.9%
Okita (2016)	12	118	36	450	1.30	[0.65; 2.59]	3.9%	4.3%
Pang (2015)	4	25	8	166	3.76	[1.04; 13.58]	0.5%	2.7%
Pepin (2009)	12	35	59	206	1.30	[0.61; 2.78]	3.3%	4.1%
Rasmussen (2009)	21	65	19	110	2.29	[1.12; 4.68]	2.8%	4.2%
Rem (2019)	24	96	11	217	6.24	[2.91; 13.38]	1.5%	4.1%
Ruttmann (2006)	11	65	19	149	1.39	[0.62; 3.13]	2.8%	3.9%
Ruttmann (2020)	17	135	40	305	0.95	[0.52; 1.75]	6.2%	4.5%
Sorabella (2015)	5	54	18	254	1.34	[0.47; 3.78]	1.7%	3.3%
Subbaraju (2018)	9	30	15	109	2.69	[1.04; 6.96]	1.3%	3.5%
Wang (2007)	34	423	93	2028	1.82	[1.21; 2.73]	8.6%	5.0%
Yeates (2010)	2	13	0	95	41.52	[1.88; 919.29]	0.0%	0.8%
Horng (1993)	7	21	5	134	12.90	[3.61; 46.09]	0.3%	2.7%
Champney (2016)	6	43	2	19	1.38	[0.25; 7.55]	0.7%	2.0%
Suzuki (2016)	5	25	2	43	5.13	[0.91; 28.76]	0.3%	1.9%
**Common effect model**		**2457**		**9340**	**1.98**	**[1.75; 2.23]**	**100.0%**	**--**
**Random effects model**					**2.35**	**[1.74; 3.16]**	**--**	**100.0%**

Heterogeneity: $I^2 = 70\%$, $\tau^2 = 0.4155$, $p < 0.01$

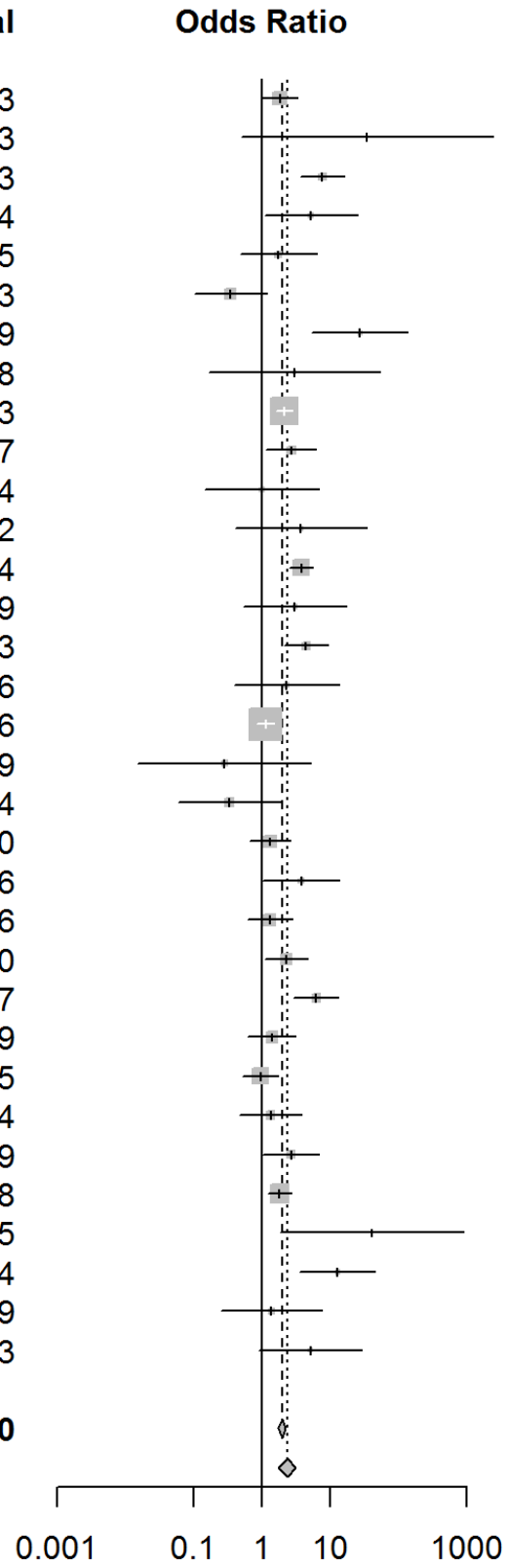

Supplementary Figure 8. Frequency of A successful reperfusion (TICI 2b or 3), B symptomatic intracerebral hemorrhage, C mortality and D good clinical outcome at discharge (mRS 0 – 2) in patients with IE and stroke undergoing mechanical thrombectomy

A

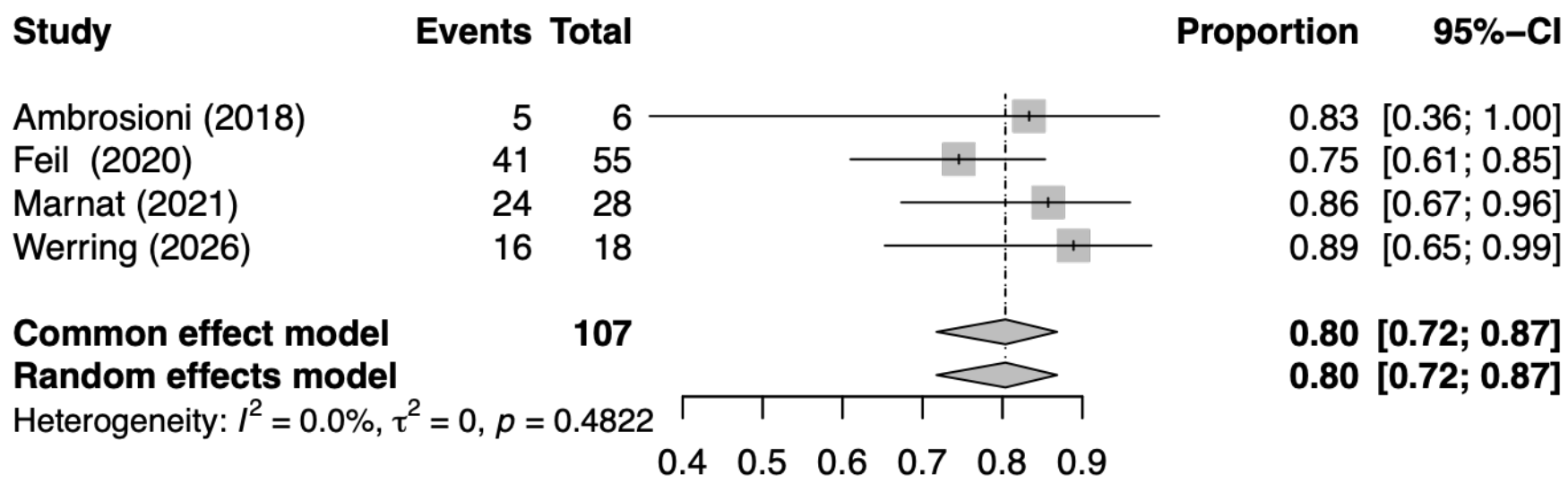

B

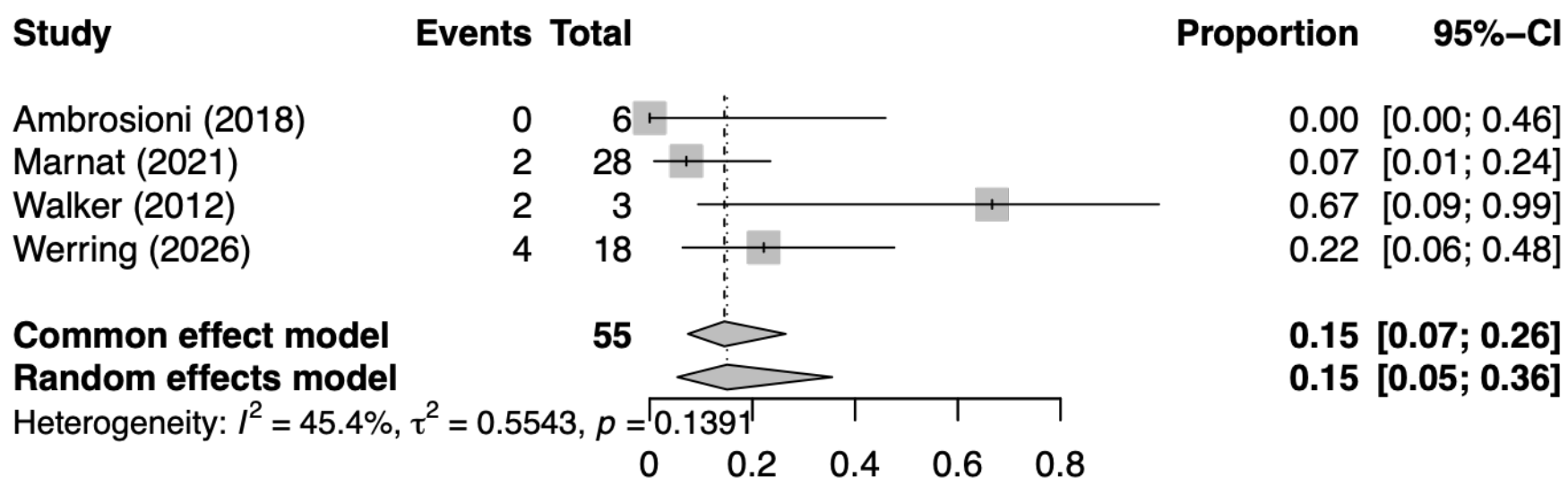

C

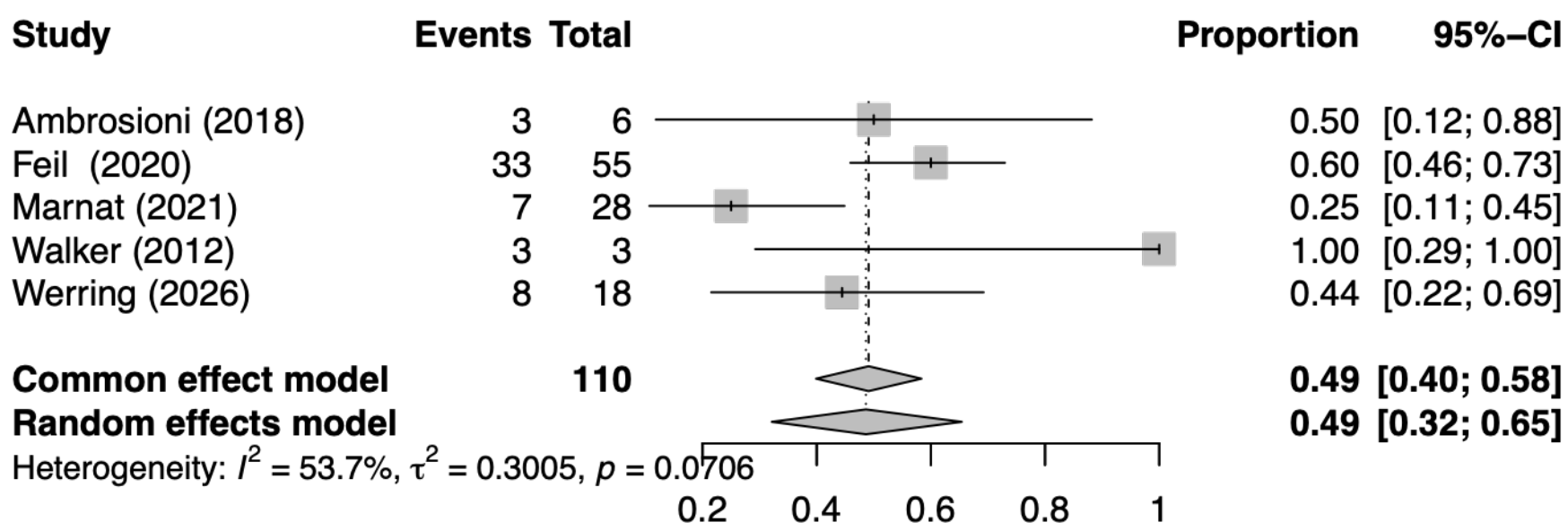

D

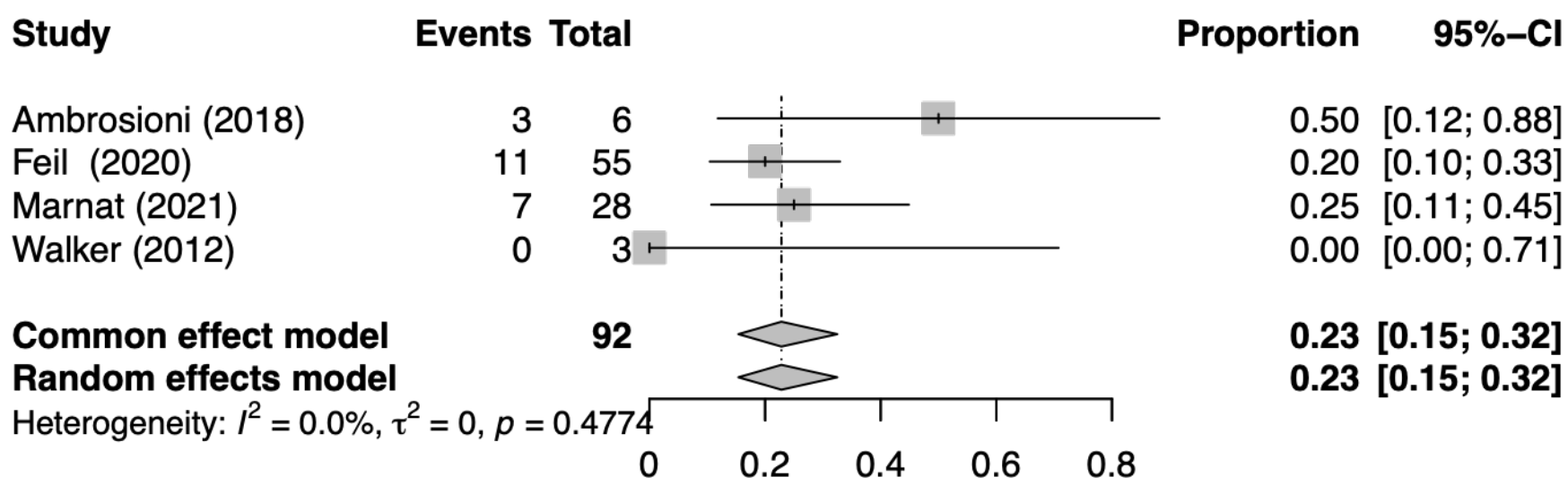

References

1. Haddaway NR, Page MJ, Pritchard CC, McGuinness LA. *PRISMA2020* : An R package and Shiny app for producing PRISMA 2020-compliant flow diagrams, with interactivity for optimised digital transparency and Open Synthesis. Campbell Syst. Rev. 2022;18(2):e1230.
